# Supplementary material for: Cullin5 drives experimental asthma exacerbations by modulating alveolar macrophage antiviral immunity
Source: Nat Commun. 2024 Jan 4;15:252. doi: 10.1038/s41467-023-44168-0 (PMC10766641; doi:10.1038/s41467-023-44168-0)
Supplement: Supplementary file 1 — Supplementary Information [file 41467_2023_44168_MOESM1_ESM.pdf]

Cullin5 drives experimental asthma exacerbations by modulating alveolar macrophage antiviral immunity

Haibo Zhang[1, 2, 3], Keke Xue[1, 2, 3], Wen Li[1, 2, 3], Xinyi Yang[1, 2, 3], Yusen Gou[1, 2, 3], Xiao Su[4], Feng Qian[1, 2, 3, *], Lei Sun[1, 2, 3, *]

[1] Shanghai Frontiers Science Center of Drug Target Identification and Delivery, School of Pharmaceutical Sciences, Shanghai Jiao Tong University, Shanghai, 200240, P. R. China

[2] National Key Laboratory of Innovative Immunotherapy, Shanghai Jiao Tong University, Shanghai, 200240, P. R. China

[3] Engineering Research Center of Cell & Therapeutic Antibody, Ministry of Education, School of Pharmaceutical Sciences, Shanghai Jiao Tong University, Shanghai, 200240, P. R. China

[4] Unit of Respiratory Infection and Immunity, Shanghai Institute of Immunity and Infection, Chinese Academy of Sciences, Shanghai, 200031, P.R. China

* **Corresponding author:** Lei Sun and Feng Qian, School of Pharmacy, Shanghai Jiao Tong University, 800 Dongchuan Road, Shanghai, 200240, P.R. China. Tel: +86-21+34207424, Fax: +86-21-34204457, E-mail: sunlei_vicky@sjtu.edu.cn and fengqian@sjtu.edu.cn

Supplementary Fig. 1

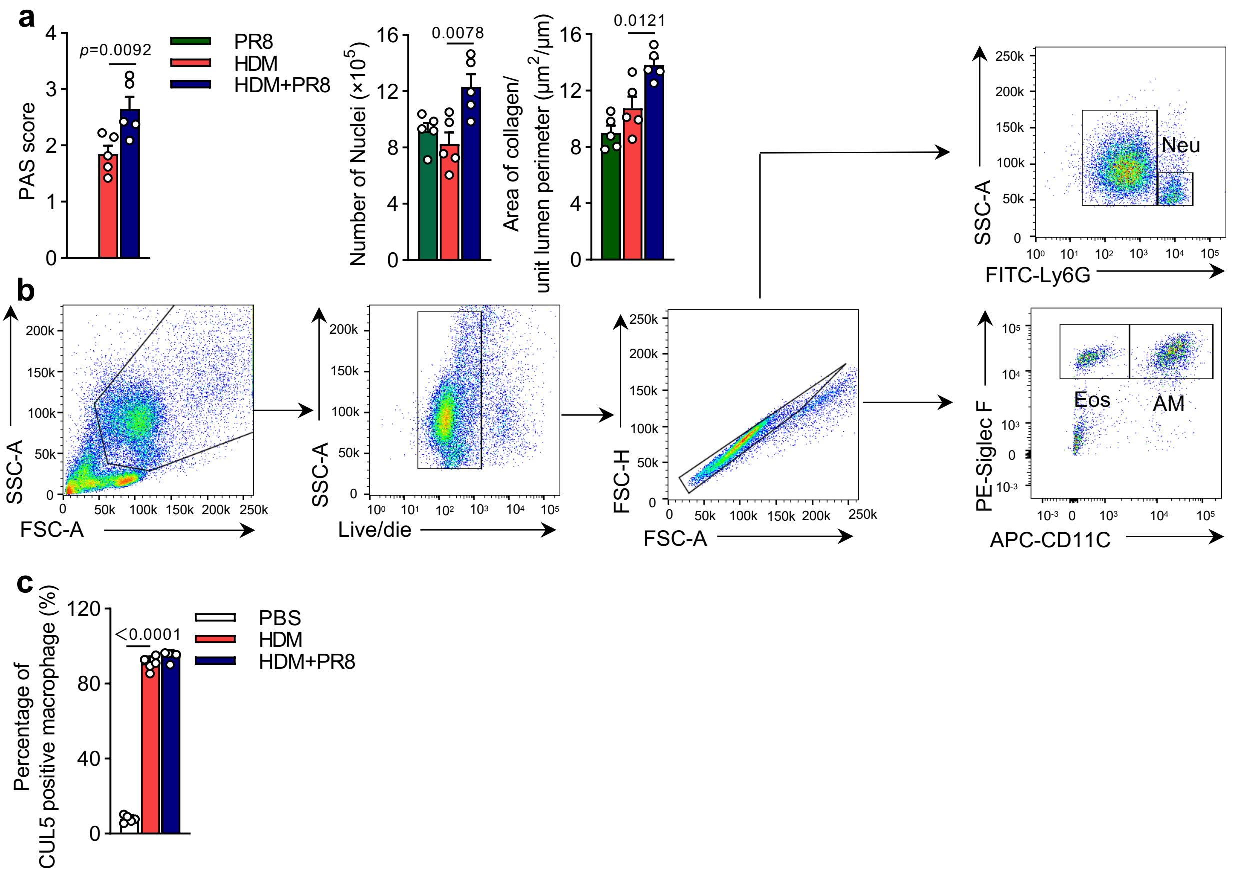

Supplementary Fig. 1 Establishment of influenza-induced asthma exacerbations animal model. a, Determination of mucus producing cell score by periodic acid-Schiff (PAS) staining, nuclei count by hematoxylin and eosin (H&E) staining, and airway collagen deposition area/unit lumen perimeter by Masson's trichome-staining in each group of mice in Fig. 1c. **b,** Gating strategies of AM ($CD11c^+$ Siglec-F^+), Neu ($Ly6G^+$) and Eos ($CD11c^-$ Siglec-F^+) in the BALF of mice in figures 1-3, 5, 8 and supplementary figures 3-5. **c**, Determination of the percentage of CUL5-positive macrophages (calculated as the ratio of CUL5-positive macrophages to total macrophages) in the whole lung tissues of mice in each group in Fig. 1 using the ImageJ software. Data are representative of three independent experiments (mean ± s.e.m.), n = 5 per group. *p*-value was calculated by one-way ANOVA (Tukey's test). Source data are provided as a Source Data file.

Supplementary Fig. 2

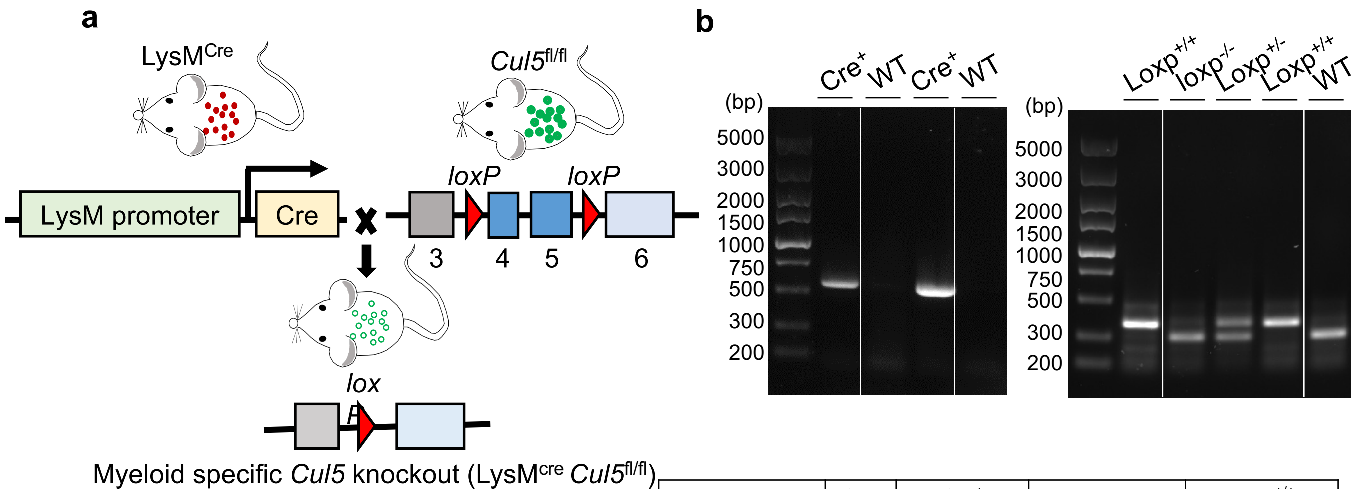

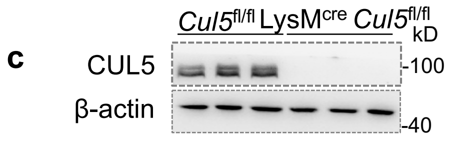

Mice type	Cre⁻ (WT)	Cre⁺ (Cre 543 bp)	WT (Cul5 247 bp)	Loxp+/+ (Neo 328 bp)
Wild type	+	-	+	-
*Cul5*fl/fl	+	-	-	+
LysMCre*Cul5*fl/fl	-	+	-	+

Supplementary Fig. 2 Establishment and identification of myeloid-specific CUL5 deficient (LysMCre *Cul5fl/fl*) mice. a, Mice conditionally containing a loxP sequence flanking the fourth and fifth exons of *Cul5* (*Cul5fl/fl*) were crossed with Lysozyme M-Cre mice (LysMCre). **b**, Mice were genotyped by PCR analysis of DNA obtained from the tail snip. Cre+ indicates Cre+/+ or Cre+/-. Flox = flanked by loxP. **c**, Immunoblot analysis of CUL5 expression in lysates of BMDMs obtained from the indicated mice. n = 3 per group. Data are representative of three independent experiments. Source data are provided as a Source Data file.

Supplementary Fig. 3

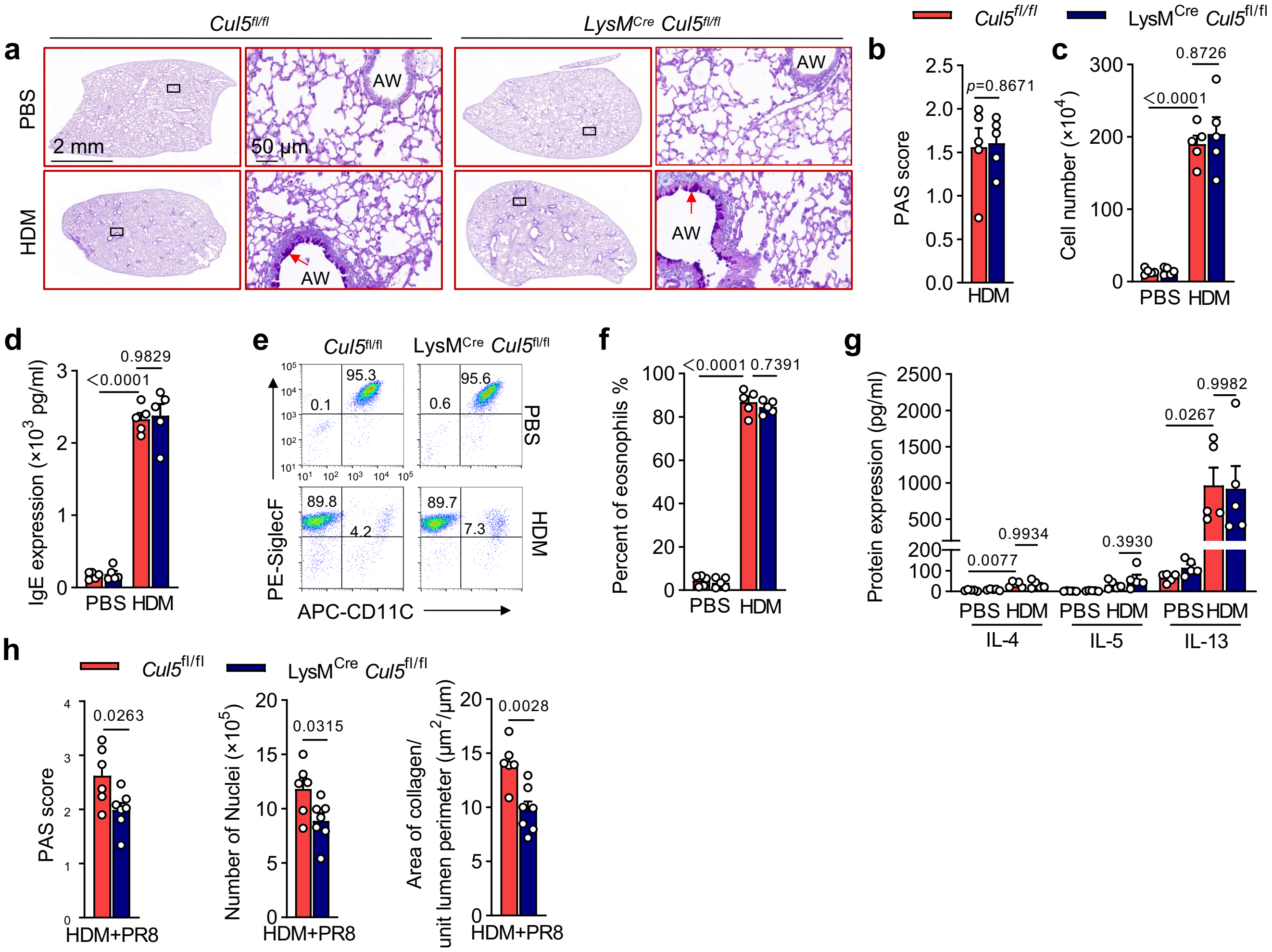

Supplementary Fig. 3 Myeloid-specific CUL5 deficiency does not influence allergic asthma pathology. a, PAS-stained lung tissues of the indicated mice. Scale bars, 2 mm and 50 µm. Red arrowheads indicate goblet cells containing mucus (magenta) after PAS staining. AW, airway. **b**, Determination of mucus producing cell score by PAS staining in the whole lung tissues of mice in each group. **c**, Total cell count in the BALF of the indicated mouse group. **d**, ELISA was performed to determine IgE serum levels in of the indicated mice. **e**, Eosinophil (Eos, $CD11c^-$ $SiglecF^+$) and alveolar macrophage (AM, $CD11c^+$ $SiglecF^+$) in BALF were analyzed by flow cytometry. **f**, Eosinophil percentage in BALF was determined. **g**, ELISA was performed to determine IL-4, IL-5, and IL-13 levels in the BALF of the indicated mice. **h**, Determination of mucus producing cell score by PAS staining, nuclei count by H&E staining, and airway collagen deposition area/unit lumen perimeter by Masson's trichome staining in each group mice in Fig. 2b. Data are representative of three independent experiments (mean ± s.e.m.). n = 5 in (**a**)–(**g**) per group per experiment. n = 5, 5, 6, and 7 in (**h**) of the PBS-administered *Cul5*$^{fl/fl}$, PBS-administered LysM^{Cre} *Cul5*$^{fl/fl}$, HDM+PR8-administered *Cul5*$^{fl/fl}$, and HDM+PR8-administered LysM^{Cre} *Cul5*$^{fl/fl}$ groups, respectively. *p*-values were calculated by two-way ANOVA (Tukey's test), or unpaired two-tailed *t*-test in (**b**) and (**h**). Source data are provided as a Source Data file.

Supplementary Fig. 4

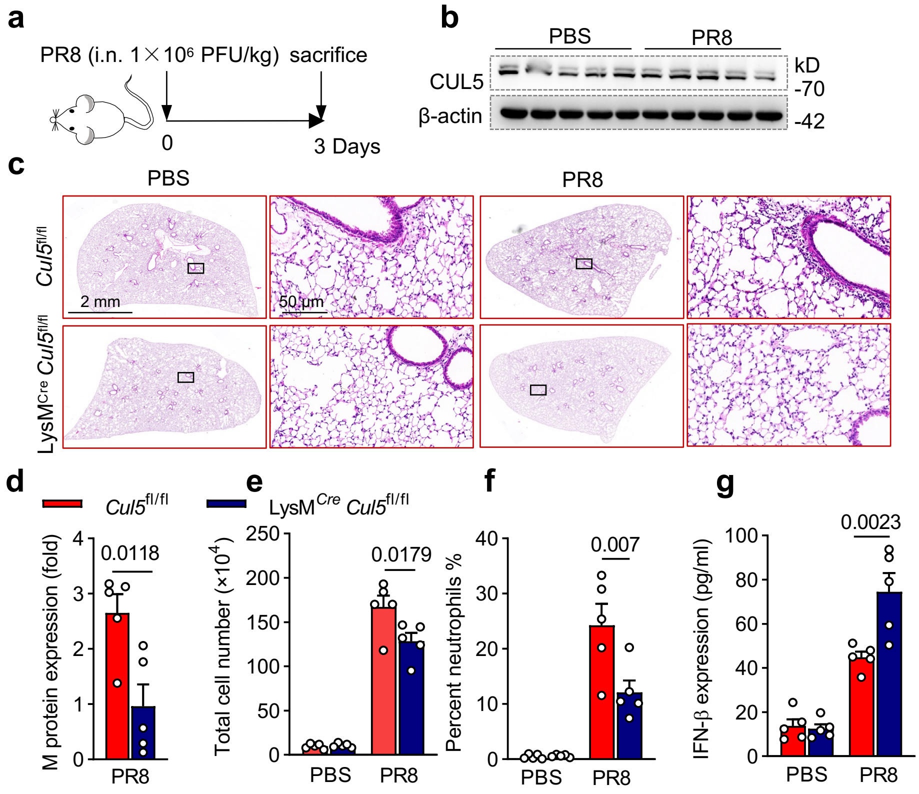

Supplementary Fig. 4 Myeloid-specific CUL5 deficiency alleviates PR8-induced pneumonia. **a**, Schemes showing the establishment of PR8-induced pneumonia. **b**, Immunoblotting of CUL5 and β-actin in lysates of lung tissue for the indicated group. **c**, H&E-stained lung tissues of the indicated mice. Scale bars, 100 µm. **d**, Relative influenza virus content in the lung tissues of the indicated groups was quantified based on M protein levels measured by RT-qPCR and shown as ΔCt fold change. **e**, Total cell count in the BALF of the indicated mouse group. **f**, Neutrophil (Ly6G^{+}) percentage in BALF was determined. **g,** ELISA was performed to determine IFN-β level in the BALF of the indicated mice. Data shown are representative of three independent experiments (mean ± s.e.m.), n = 5 per group per experiment. *p*-values in (**d**) were calculated by unpaired two-tailed t-test, or two-way ANOVA (Tukey's test) in (**e**)-(**g**). Source data are provided as a Source Data file.

Supplementary Fig. 5

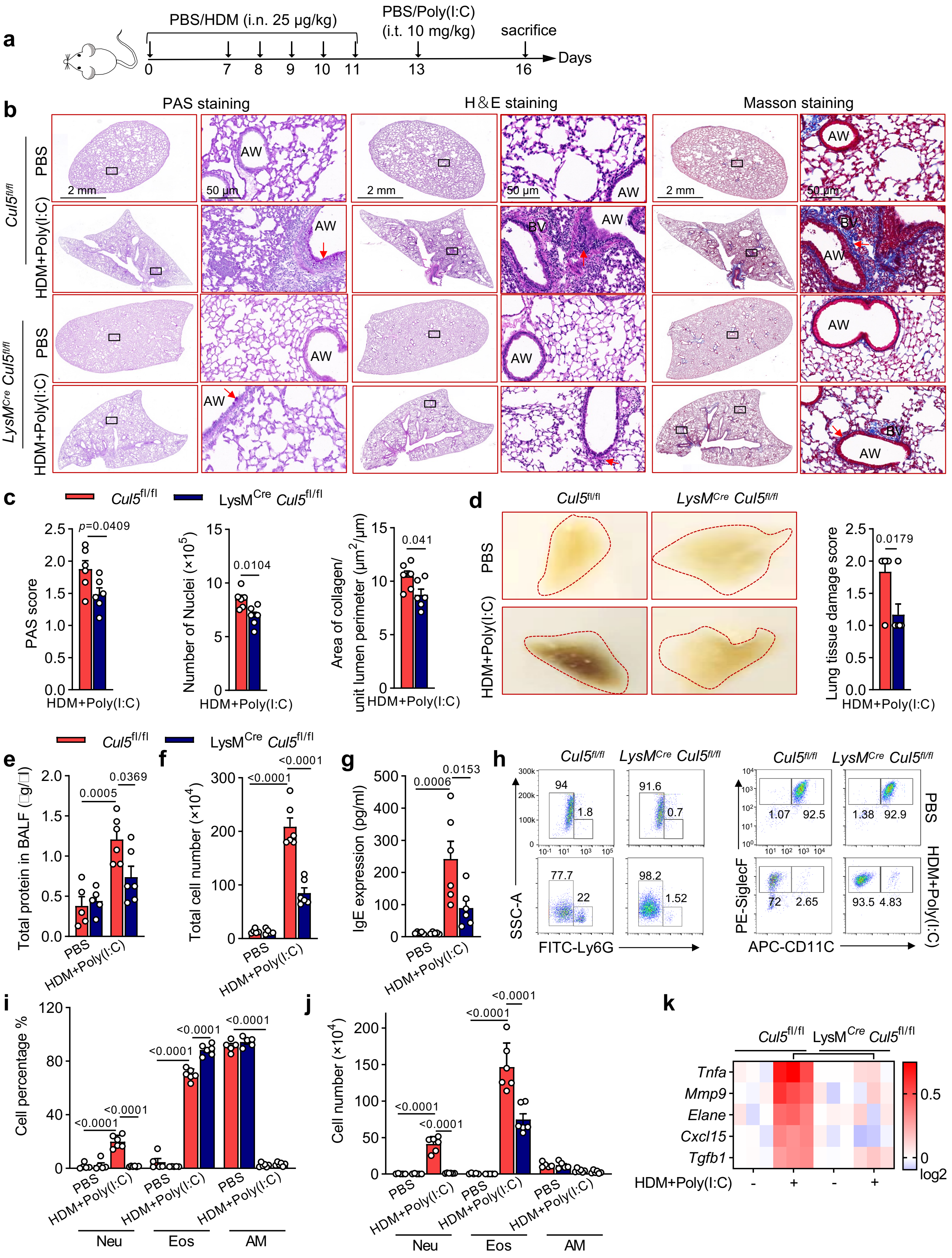

Supplementary Fig. 5 Myeloid-specific CUL5 deficiency alleviates asthma exacerbations induced by HDM combined with Poly(I:C). a, Schemes of asthma exacerbations induced by HDM combined with Poly(I:C). **b**, Periodic acid-Schiff (PAS), hematoxylin and eosin (H&E), and Masson's trichrome-stained lung tissues of the indicated mice. Red arrowheads indicate goblet cells containing mucus (magenta) after PAS staining, inflammatory infiltration after H&E staining, and collagenous fibers after Masson's trichome staining. AW, airway; BV, blood vessel. Scale bars, 2 mm and 50 μm. **c**, Determination of mucus producing cell score by PAS staining, nuclei count by H&E staining, and airway collagen deposition area/unit lumen perimeter of by Masson's trichome staining in each group mice in (**b**). **d,** Paraffin-embedded lung tissue of the indicated group of mice. Dark-brown lung tissue indicates severe capillary rupture. Tissue damage was quantified using the ImageJ software. **e**, BCA test was performed to determine total protein levels in the BALF of the indicated mice. **f**, Total BALF cell counts were determined in the indicated mice. **g**, ELISA was performed to determine IgE serum levels in the indicated mice. **h**, Neutrophil (Neu, $Ly6G^+$), eosinophil (Eos, $CD11c^-$ $SiglecF^+$), and alveolar macrophage (AM, $CD11c^+$ $SiglecF^+$) in BALF were analyzed by flow cytometry. **i–j**, Percentages and counts of Neu, Eos, and AM in BALF were determined. **k**, Heatmap summarizing the mRNA expression of *Tnfa, Mmp9, Cxcl15, Elane,* and *Tgfb* in the lung tissues of the indicated mice. Data are representative of three independent experiments (mean ± s.e.m.), n = 5, 5, 6, and 6 in the PBS-administered *Cul5*$^{fl/fl}$, PBS-administered LysM^{Cre} *Cul5*$^{fl/fl}$, HDM+Poly(I:C)-administered *Cul5*$^{fl/fl}$, and HDM+Poly(I:C)-administered LysM^{Cre} *Cul5*$^{fl/fl}$ groups, respectively. *p*-values were calculated by unpaired two-tailed *t*-test (**c**) and (**d**), or two-way ANOVA (Tukey's test) (**e**)-(**k**). Source data are provided as a Source Data file.

Supplementary Fig. 6

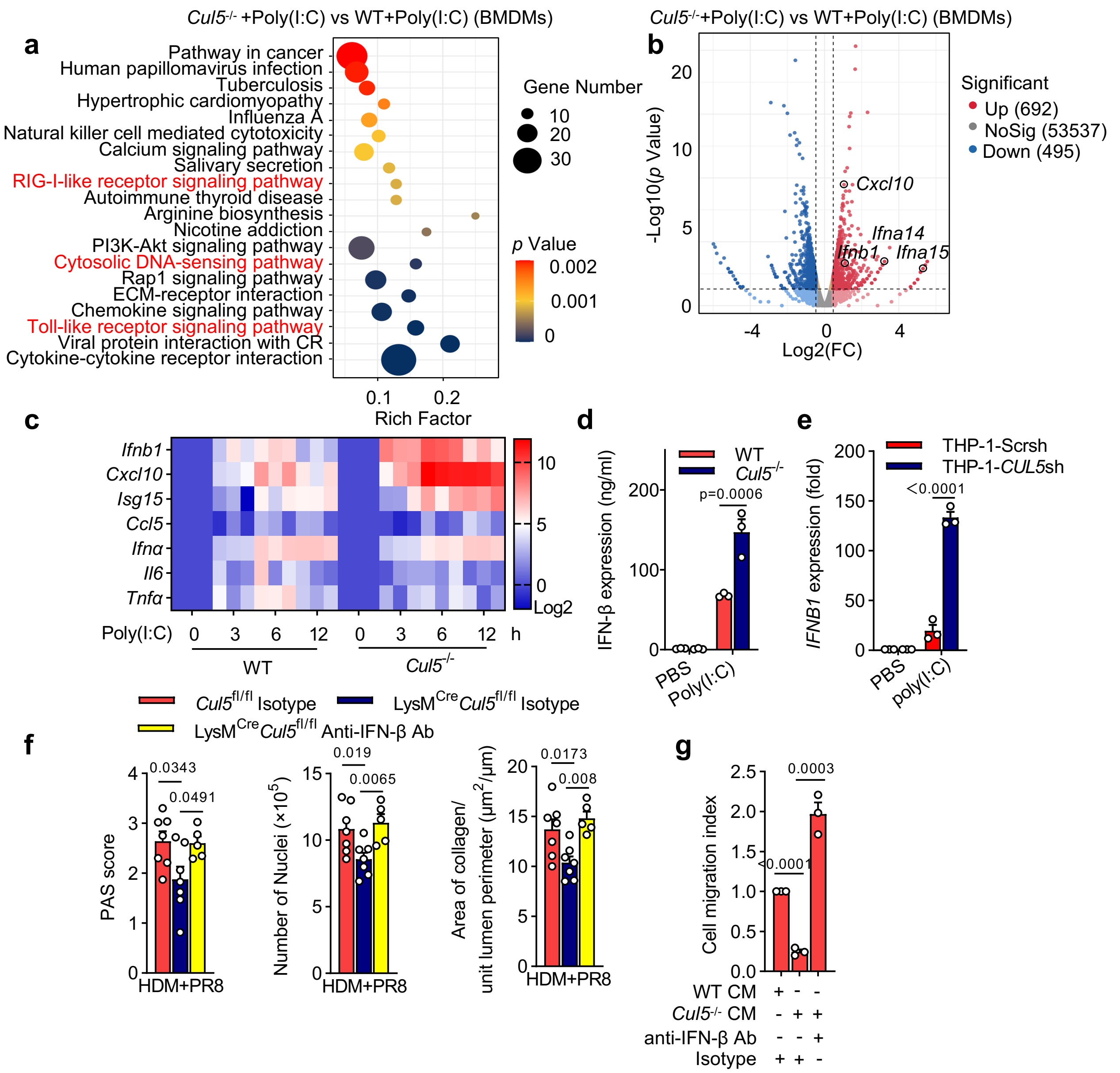

Supplementary Fig. 6 CUL5 promotes asthma exacerbations and neutrophil migration via IFN-β inhibition. a, Dot graph showing the top 20 KEGG enriched pathways in DEGs between BMDMs isolated from *Cul5*$^{fl/fl}$ (WT) and LysM^{Cre} *Cul5*$^{fl/fl}$ (*Cul5*$^{-/-}$) mice and treated with 4 μg/ml Poly(I:C) for 6 h, with pattern recognition receptor-related pathways highlighted in red. *p*-value was calculated by Fisher's exact test. **b**, Volcano plots of DEGs in CUL5-deficient BMDMs and WT BMDMs treated with 4 μg/ml Poly(I:C) for 6 h, with interferon and interferon-associated genes, *Cxcl10, Ifna14, Ifna15,* and *Ifnb1* marked. DEGs were defined using the following criteria: Log2 (foldchange) <–1 or >1 and padj <0.05. **c**, Heatmap summarizing the mRNA expression of *Ifnb*, *Cxcl10*, *Isg15*, *Ccl5*, *Il6*, and *Tnfa* in BMDMs stimulated with 4 μg/ml Poly(I:C) for indicated times. n = 3 biological replicates. **d**, ELISA was performed to determine IFN-β concentration in the culture medium of BMDMs treated with 4 μg/ml Poly(I:C) for 12 h. **e**, qPCR was performed to determine *IFNB1* mRNA level in PMA-induced THP-1 macrophages treated with 4 μg/ml Poly(I:C) for 6 h. **f**, Determination of mucus producing cell score by PAS staining, nuclei count by H&E staining, and airway collagen deposition area/unit lumen perimeter by Masson's trichome staining in each group mice in Fig. 3e. Data are representative of three independent experiments (mean ± s.e.m.), n = 7, 7, and 5 in the HDM+PR8-administered *Cul5*$^{fl/fl}$, HDM+PR8-administered LysM^{Cre} *Cul5*$^{fl/fl}$, and HDM+PR8+anti-IFN-β-administered LysM^{Cre} *Cul5*$^{fl/fl}$ groups, respectively. **g**, The cell migration index represents the total area of migrating cells in the upper and lower chambers of the corresponding group divided by that of the WT CM group in Fig. 3m. Data represent the mean ± s.e.m., n = 3 biological replicates. *p*-values in (**c**) - (**e**) were calculated by two-way ANOVA (Tukey's test), unpaired two-tailed *t*-test in (**f**), or one-way ANOVA (Tukey's test) in (**g**). Source data are provided as a Source Data file.

Supplementary Fig. 7

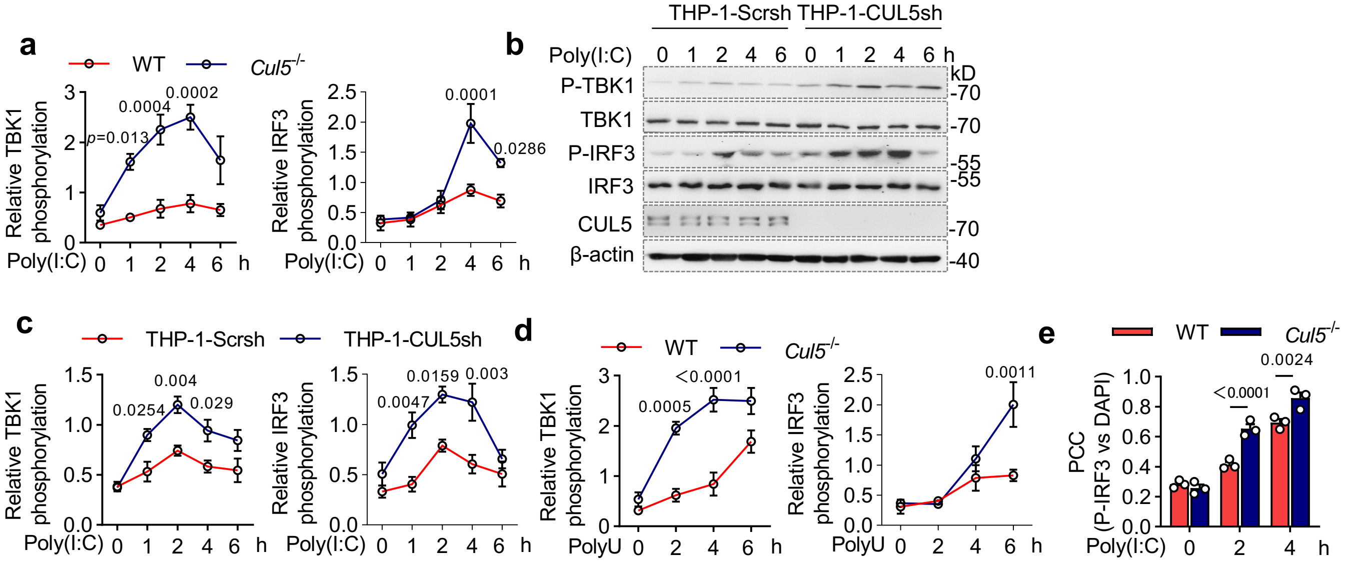

Supplementary Fig. 7 CUL5 deficiency upregulated IFN-β expression through improving RIG-1-like receptors (RLRs) signaling pathway. a, Densitometric analysis of the immunoblot of phosphorylated (P-)TBK1 and P-IRF3 shown in Fig. 4a. **b**, Immunoblot of phosphorylated (P-)TBK1, TBK1, P-IRF3, IRF3, and β-actin in lysates of PMA-induced THP-1-CUL5sh or THP-1-Scrsh macrophages stimulated by 4 μg/ml Poly(I:C) for the indicated times. **c**, Densitometric analysis of the immunoblot of (P-)TBK1 and P-IRF3 shown in (**b**). **d**, Densitometric analysis of the immunoblot of (P-)TBK1 and P-IRF3 shown in Fig. 4b. **e**, Pearson's correlation coefficients in Fig. 4c were determined to evaluate the correlation of voxel intensity between the DAPI (blue) and P-IRF3 (red) channels. Data are representative of three independent experiments, or represent the mean ± s.e.m., n = 3 biological replicates. *p*-value were calculated by two-way ANOVA (Sidak's test). Source data are provided as a Source Data file.

Supplementary Fig. 8

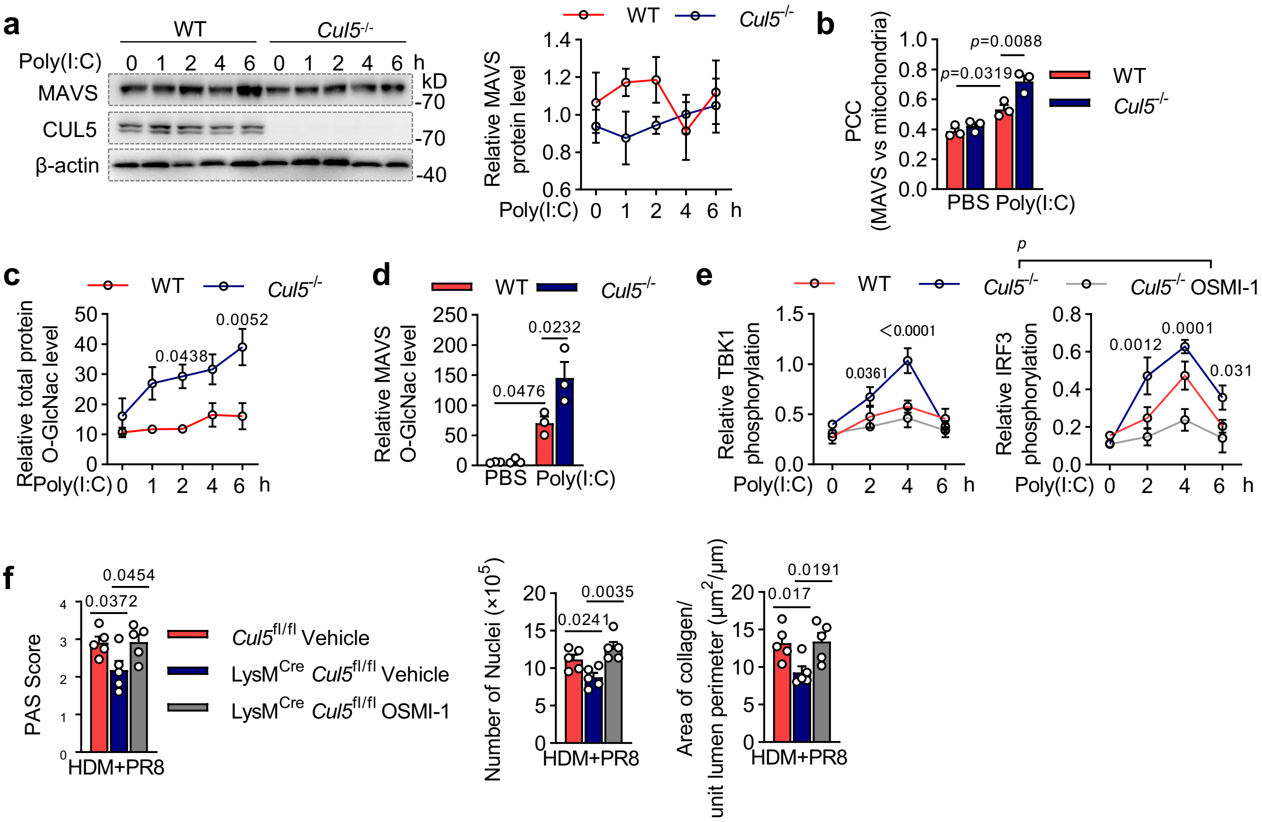

Supplementary Fig. 8 CUL5 in alveolar macrophages (AMs) regulates virus-induced asthma exacerbations via O-GlcNAc transferase (OGT). a, Immunoblot analysis of mitochondrial antiviral-signaling protein (MAVS) in lysates of BMDMs stimulated by 4 μg/ml Poly(I:C) for indicated times. Densitometric analysis of the immunoblot of MAVS. **b**, Pearson's correlation coefficients in Fig. 4f were determined to evaluate the correlation of voxel intensity between the mitochondrial (red) and MAVS (green) channels. **c**, Densitometric analysis of the immunoblot of total O-GlcNac protein levels shown in Fig. 4i. **d**, Densitometric analysis of the immunoblot of MAVS O-GlcNac protein levels shown in Fig. 4j. **e**, Densitometric analysis of the immunoblot of phosphorylated (P-)TBK1 and P-IRF3 shown in Fig. 4l. **f**, Determination of mucus producing cell score by PAS staining, nuclei counts by hematoxylin and eosin H&E staining, and airway collagen deposition area/unit lumen perimeter by Masson's trichome staining in each group mice in Fig. 5e. Data are representative of three independent experiments, or represent the mean ± s.e.m., n = 3 biological replicates. *p*-value were calculated by two-way ANOVA (Sidak's test) in (**a**) and (**c**), or (Tukey's test) in (**b**), (**d**), and (**e**), or unpaired two-tailed t-test in (**f**). Source data are provided as a Source Data file.

Supplementary Fig. 9

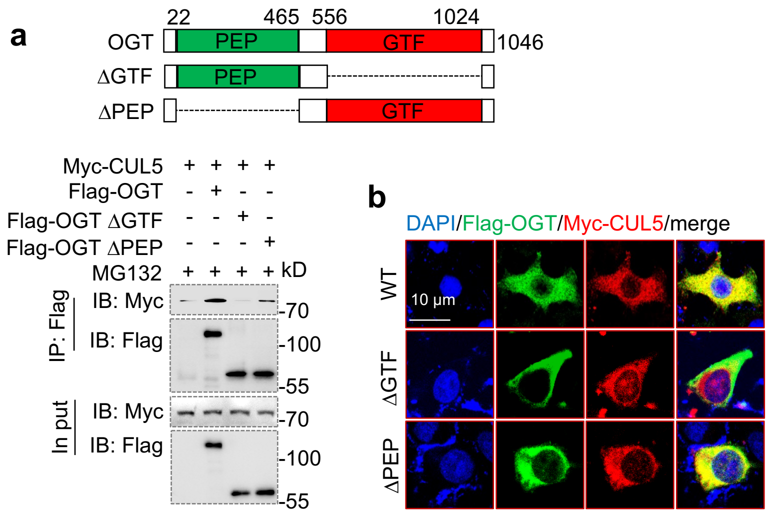

Supplementary Fig. 9 CUL5 interacts with O-GlcNAc transferase (OGT) via the GTF domain. a, Co-immunoprecipitation (Co-IP) and immunoblot analyses of HEK293T cells co-transfected with Flag-OGT, Myc-CUL5, and Flag-OGT mutants including Flag-OGT ΔGTF (glycosyl transferase family domain deletion) and Flag-OGT ΔPEP (PEP-CTERM system TPR-repeat lipoprotein domain deletion). **b**, Confocal microscopic imaging of HeLa cells co-transfected with Myc-CUL5 (red), Flag-OGT (WT), and Flag-OGT mutants (green). Nuclei were stained with DAPI (blue). Yellow in merge indicates the co-localization of Myc-CUL5 and Flag-OGT. Scale bars, 10 μm. Data are representative of three independent experiments. Source data are provided as a Source Data file.

Supplementary Fig. 10

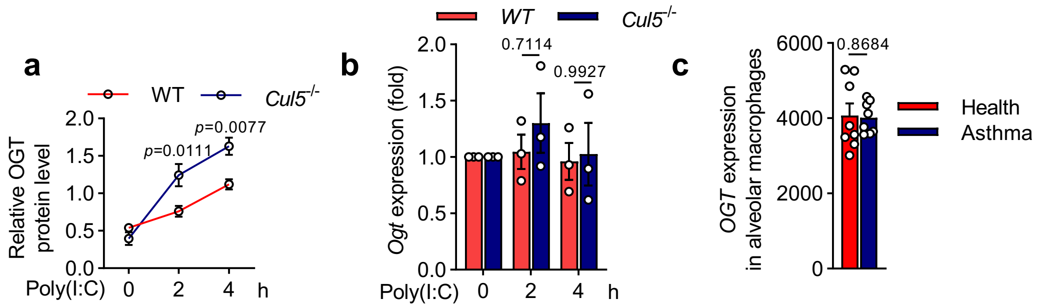

Supplementary Fig. 10 CUL5 regulates O-GlcNAc transferase (OGT) protein expression. a, Densitometric analysis of the immunoblot of OGT protein levels shown in Fig. 6d. **b**, Relative mRNA level of *Ogt* in BMDMs treated with 4 μg/ml Poly(I:C) for the indicated times. **c**, Relative expression of *OGT* in alveolar macrophages (AMs) from the GEO database (GSE6858). n = 8 healthy persons and 8 patients with asthma. Data represent the mean ± s.e.m., n = 3 biological replicates. *p*-value in (**a**) and (**b**) were calculated by two-way ANOVA (Sidak's test), or unpaired two-tailed t-test in (**c**). Source data are provided as a Source Data file.

Supplementary Fig. 11

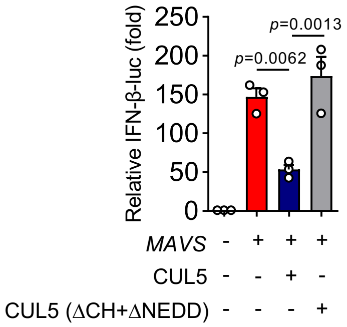

Supplementary Fig. 11 CUL5 inhibits MAVS induced IFN-β-luciferase reporter activation via the CH and NEDD domains. Dual-luciferase reporter system analysis of relative IFN-β-luc activation based on pRL-TK-luc with the indicated co-transfected plasmids. Data represent the mean ± s.e.m. n = 3 biological replicates. *p*-value was calculated by one-way ANOVA (Tukey's test). Source data are provided as a Source Data file.

Supplementary Fig. 12

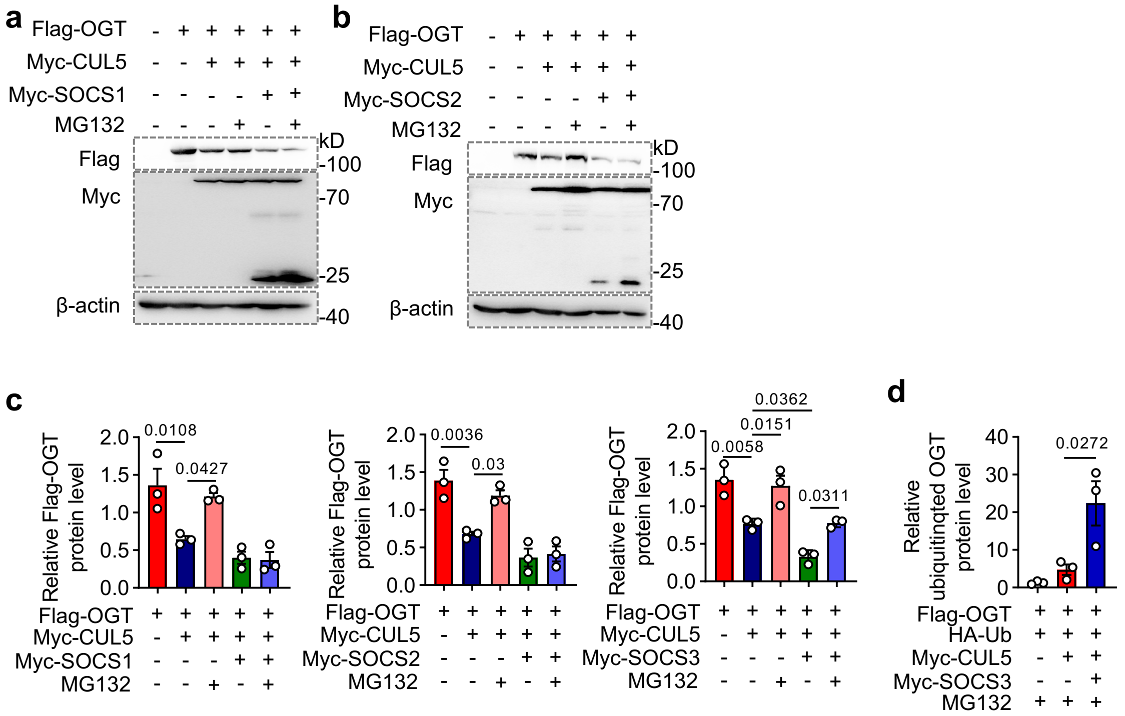

Supplementary Fig. 12 CUL5 promote O-GlcNAc transferase (OGT) degradation via SOCS3. a-b, Immunoblot analysis indicating protein expression in lysates of HEK293T cells co-transfected with Flag-OGT, Myc-CUL5, and Myc-SOCS1 (**a**), or Myc-SOCS2 (**b**). Data are representative of three independent experiments. **c**, Densitometric analysis of the immunoblot of Flag-OGT protein levels shown in Fig. 6l and Supplementary Fig 11a-b. **d,** Densitometric analysis of the immunoblot of ubiquitinated OGT protein levels shown in Fig. 6n. Data represent the mean ± s.e.m., n = 3 biological replicates. *p*-values were calculated by one-way ANOVA (Tukey's test). Source data are provided as a Source Data file.

Supplementary Fig. 13

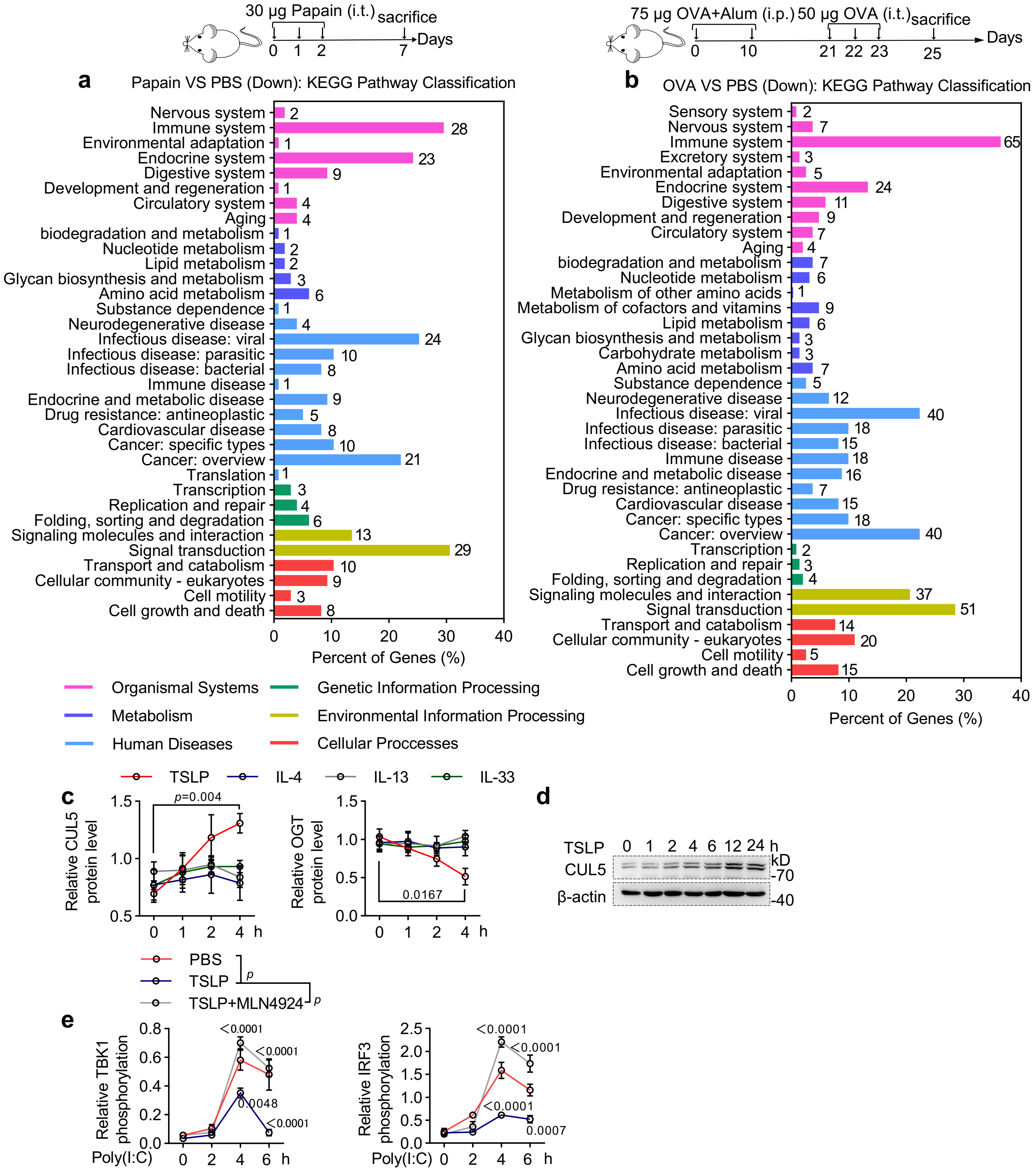

Supplementary Fig. 13 Thymic stromal lymphopoietin (TSLP) regulates CUL5-mediated viral susceptibility in allergic asthma mice. Schemes and further classification of downregulated enriched pathways in the dot graph disrupted by (**a**) papain or (**b**) OVA treatment. The horizontal axis represents the ratio of the total number of downregulated differentially expressed genes (DEGs) annotated to each level 2 pathway to the total number of downregulated DEGs annotated to the KEGG pathway (%). The vertical axis represents the name of the level 2 pathway. The numbers to the right of the column represent the number of downregulated DEGs annotated to the corresponding level 2 pathway. **c**, Densitometric analysis of the immunoblot of CUL5 and OGT protein levels shown in Fig. 7b. **d,** BMDMs were treated by 20 μM TSLP for indicated time. The protein level of CUL5 was detected by immunoblot. Data are representative of three independent experiments. **e,** Densitometric analysis of the immunoblot of phosphorylated (P-)TBK1 and P-IRF3 shown in Fig. 7h. Data represent the mean ± s.e.m., n = 3 biological replicates. *p*-value were calculated by one-way ANOVA (Tukey's test) in (**c**), or two-way ANOVA (Tukey's test) in (**e**). Source data are provided as a Source Data file.

Supplementary Fig. 14

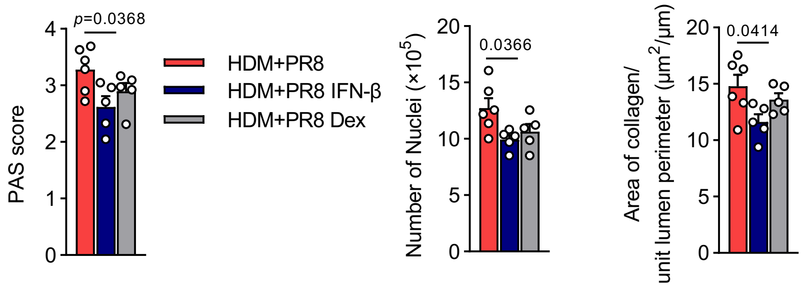

Supplementary Fig. 14 IFN-β treatment induces remission of neutrophilic asthma exacerbations. Determination of mucus producing cell score by PAS staining, nuclei count by H&E staining, and airway collagen deposition area/unit lumen perimeter by Masson's trichome staining in each group mice in Fig. 8d. Data represent the mean ± s.e.m. of one out of three independent experiments. n = 5, 6, 5, and 5 in the PBS, HDM+PR8, HDM+PR8 IFN-β, and HDM+PR8 Dex groups, respectively. *p*-values were calculated by one-way ANOVA (Tukey's test). Source data are provided as a Source Data file.

Supplementary Table 1

Peptide	Position	Score	Threshold
HFSTLAIKQNPLLAE	83	0.93	0.3
SDLGNLLKALGRLEE	168	1.06	0.3
LGRLEEAKACYLKAI	177	1.28	0.3
EAKACYLKAIETQPN	182	1.05	0.3
LAIHHFEKAVTLDPN	216	0.88	0.3
LANALKEKGSVAEAE	306	2.85	0.3
EAVRLYRKALEVFPE	352	0.66	0.3
SVLQQQGKLQEALMH	376	0.39	0.3
ASYRTALKLKPDFPD	457	0.65	0.3
YRTALKLKPDFPDAY	459	1.82	0.3
PLSHGFRKAIAERHG	521	1.18	0.3
HGNLCLDKINVLHKP	534	0.64	0.3
KPPYEHPKDLKLSDG	547	1.45	0.3
YEHPKDLKLSDGRLR	550	1.8	0.3
SQIPCNGKAADRIHQ	623	0.77	0.3
VNMNGYTKGARNELF	644	0.31	0.3
ANMFPHLKKKAVIDF	716	0.42	0.3
NMFPHLKKKAVIDFK	717	0.99	0.3
MFPHLKKKAVIDFKS	718	1.11	0.3
VLNGIDLKAFLDSLP	742	0.31	0.3
LPDVKIVKMKCPDGG	755	0.84	0.3
ATTQINNKAATGEEV	816	0.96	0.3
GTDLEYLKKVRGKVW	991	1.36	0.3
YLKKVRGKVWKQRIS	996	1.65	0.3
KVRGKVWKQRISSPL	999	1.47	0.3
NMFPHLKKKAVIDFK	717	0.99	0.3
MFPHLKKKAVIDFKS	718	1.11	0.3
ATTQINNKAATGEEV	816	0.96	0.3
GTDLEYLKKVRGKVW	991	1.36	0.3
YLKKVRGKVWKQRIS	996	1.65	0.3
KVRGKVWKQRISSPL	999	1.47	0.3

Supplementary Table 1: Potential ubiquitination sites.

Supplementary Table 2

Names	Cat	Company	Dilution	Link
RIG-I Ab	4200S	Cell Signaling Technology	1:1000	https://www.cellsignal.cn/products/primary-antibodies/rig-i-d33h10-rabbit-mab/4200
MAVS Ab	83000S	Cell Signaling Technology	1:100 for IP and 1:1000 for western blot	https://www.cellsignal.cn/products/primary-antibodies/mavs-e8z7m-rabbit-mab/83000
Phospho-TBK1/NAK Ab,Ser172	5483S	Cell Signaling Technology	1:1000	https://www.cellsignal.cn/products/primary-antibodies/phospho-tbk1-nak-ser172-d52c2-xp-rabbit-mab/5483
TBK1/NAK Ab	38066S	Cell Signaling Technology	1:1000	https://www.cellsignal.cn/products/primary-antibodies/tbk1-nak-e8i3g-rabbit-mab/38066
Phospho-IRF-3 Ab, Ser396	29047S	Cell Signaling Technology	1:1000	https://www.cellsignal.cn/products/primary-antibodies/phospho-irf-3-ser396-d6o1m-rabbit-mab/29047
IRF-3 Ab	4302S	Cell Signaling Technology	1:1000	https://www.cellsignal.cn/products/primary-antibodies/irf-3-d83b9-rabbit-mab/4302
β-actin Ab	4970T	Cell Signaling Technology	1:1000	https://www.cellsignal.cn/products/primary-antibodies/b-actin-13e5-rabbit-mab/4970
SOCS3 Ab	52113S	Cell Signaling Technology	1:1000	https://www.cellsignal.cn/products/primary-antibodies/socs3-d6e1t-rabbit-mab/52113
SOCS2 Ab	2779	Cell Signaling Technology	1:1000	https://www.cellsignal.cn/products/primary-antibodies/socs2-antibody/2779
SOCS1 Ab	3950T	Cell Signaling Technology	1:1000	https://www.cellsignal.cn/products/primary-antibodies/socs1-a156-antibody/3950
Ubiquitin Ab	3936S	Cell Signaling Technology	1:1000	https://www.cellsignal.cn/products/primary-antibodies/ubiquitin-p4d1-mouse-mab/3936
F4/80 Ab	71299S	Cell Signaling Technology	1:1000	https://www.cellsignal.cn/products/primary-antibodies/f4-80-bm8-1-rat-mab/71299
HA-Tag Ab	3724S	Cell Signaling Technology	1:100 for IP and 1:1000 for western blot	https://www.cellsignal.cn/products/primary-antibodies/ha-tag-c29f4-rabbit-mab/3724
Myc-Tag Ab	2272S	Cell Signaling Technology	1:100 for IP and 1:1000 for western blot	https://www.cellsignal.cn/products/primary-antibodies/myc-tag-antibody/2272
O-GlcNAc transferase Ab	24083S	Cell Signaling Technology	1:1000	https://www.cellsignal.cn/products/primary-antibodies/ogt-d1d8q-rabbit-mab/24083
O-GlcNAc transferase Ab	sc-74546	Santa Cruz Biotechnology	1:200 for WB and 1:50 for IP/IF	https://www.scbt.com/p/o-glcnac-transferase-antibody-f-12?requestFrom=search
O-GlcNac Ab	sc-59623	Santa Cruz Biotechnology	1:200 for WB and 1:50 for IP/IF	https://www.scbt.com/p/o-glcnac-antibody-ctd110-6?requestFrom=search
CUL5 Ab	sc373822	Santa Cruz Biotechnology	1:200 for WB and 1:50 for IP/IF	https://www.scbt.com/p/cul-5-antibody-f-6?requestFrom=search
Goat anti-rabbit IgG HRP Ab	111-035-144	Jackson ImmunoResearch, Inc	1:500	https://www.jacksonimmuno.com/catalog/products/111-035-144
goat anti-mouse IgG HRP Ab	115-035-003	Jackson ImmunoResearch, Inc	1:500	https://www.jacksonimmuno.com/catalog/products/115-035-003
anti-Flag mIgG1 antibody	GM-30726AB	Genomeditech (Shanghai) Co., Ltd	1:100	https://www.ddxcell.cn/pro_details.php?class_id=102101118101103&id=1033
APC anti-mouse CD11c Ab	117310	BioLegend	1:200	https://www.biolegend.com/en-us/products/apc-anti-mouse-cd11c-antibody-1813
PE anti-mouse CD170 Ab	155506	BioLegend	1:200	https://www.biolegend.com/en-us/products/pe-anti-mouse-cd170-siglec-f-antibody-16372
FITC anti-mouse Ly6G Ab	127606	BioLegend	1:200	https://www.biolegend.com/en-us/products/fitc-anti-mouse-ly-6g-antibody-4775
APC anti-Ly6G Ab	127614	BioLegend	1:200	https://www.biolegend.com/en-us/products/apc-anti-mouse-ly-6g-antibody-6115
anti-mouse CD16/32 Ab	156603	BioLegend	1:100	https://www.biolegend.com/en-us/products/trustain-fcx-plus-anti-mouse-cd16-32-antibody-17085

Supplementary Table 2: Antibodies information.

Supplementary Table 3

Gene	Primer sequence	
	Forward (5' -> 3’)	Reverse (5' -> 3’)
Ifnb1	GCCTTTGCCATCCAAGAGATGC	ACACTGTCTGCTGGTGGAGTTC
Ifng	CAGCAACAGCAAGGCGAAAAAGG	TTTCCGCTTCCTGAGGCTGGAT
Cxcl10	ATCATCCCTGCGAGCCTATCCT	GACCTTTTTTGGCTAAACGCTTTC
Cxcl15	GGTGATATTCGAGACCATTTACTG	GCCAACAGTAGCCTTCACCCAT
Mmp9	GCTGACTACGATAAGGACGGCA	TAGTGGTGCAGGCAGAGTAGGA
Isg15	CATCCTGGTGAGGAACGAAAGG	CTCAGCCAGAACTGGTCTTCGT
Ccl5	CCTGCTGCTTTGCCTACCTCTC	ACACACTTGGCGGTTCCTTCGA
Tgfb1	TGATACGCCTGAGTGGCTGTCT	CACAAGAGCAGTGAGCGCTGAA
Tslp	GCAAATCGAGGACTGTGAGAGC	TGAGGGCTTCTCTTGTTCTCCG
Mif	GAACCGCAACTACAGTAAGCTGC	ACGTTGGCAGCGTTCATGTCGT
Il33	CTACTGCATGAGACTCCGTTCTG	AGAATCCCGTGGATAGGCAGAG
Cdhr3	CAGCACAAAAGCCAAGGCTCTTG	CCATGCAGATGTGGAGTACACG
Ccl2	GCTACAAGAGGATCACCAGCAG	GTCTGGACCCATTCCTTCTTGG
Il4	ATCATCGGCATTTTGAACGAGGTC	ACCTTGGAAGCCCTACAGACGA
Il5	GATGAGGCTTCCTGTCCCTACT	TGACAGGTTTTGGAATAGCATTTCC
Il13	AACGGCAGCATGGTATGGAGTG	TGGGTCCTGTAGATGGCATTGC
M protein	ACAGAGACTTGAAGATGTC	TCTTTAGCCATTCCATGAG
Il6	TACCACTTCACAAGTCGGAGGC	CTGCAAGTGCATCATCGTTGTTC
Tnfa	GGTGCCTATGTCTCAGCCTCTT	GCCATAGAACTGATGAGAGGGAG
Ogt	GGCTATGTGAGTTCTGACTTCGG	GATTGGCTTCCGCCATCACCTT
Elane	CAGGAACTTCGTCATGTCAGCAG	AGCCATTCTCGAAGATCCGCTG
Il1b	TGGACCTTCCAGGATGAGGACA	GTTCATCTCGGAGCCTGTAGTG
Il17a	CAGACTACCTCAACCGTTCCAC	TCCAGCTTTCCCTCCGCATTGA
Gapdh	CATCACTGCCACCCAGAAGACTG	ATGCCAGTGAGCTTCCCGTTCAG
hOGT	AGAGGCAGTTCGCTTGTATCG	GTAGGCATCAGCAAAGGTAGGAC
hOGT ΔGTF	CAAGGGTTAGCTGAGTTGGCACATC	GCGATATCGCCTACAGCCGACCATCACTGAGC
hOGT ΔPEP	AGCATCAGGAAAATCAGGCTTAAGTT	GCGATATCGCCTACCACATCTGTAGATAGAGCCGCTC
hOGT(K306R)	CTTGGTACCGAGCTCGGATCCGCCACCATGGCGTCTTCCGTGG	CCCCTCTCTTTGAGAGCATTGGCTAGGTTGCAGTAAGCATC
	CTCTCAAAGAGAGGGGCAGTGTTGCTGAAGCAGAAGATTGTT	GTCATGGTCTTTGTAGTCCTCGAGTGCTGACTCAGTGACTTCAACAGGC
hOGT(K459R)	CTTGGTACCGAGCTCGGATCCGCCACCATGGCGTCTTCCGTGG	CAGGAAAATCAGGCCTAAGTTTCAGAGCCGTGCGGT
	AGGCCTGATTTTCCTGATGCTTATTGTAACTTGGCTCATTGCC	GTCATGGTCTTTGTAGTCCTCGAGTGCTGACTCAGTGACTTCAACAGGC
hOGT(K547R)	CTTGGTACCGAGCTCGGATCCGCCACCATGGCGTCTTCCGTGG	TGAGCTTCAAGTCTCTTGGATGTTCATATGGTGGTTTATGAAGAACA
	AAGAGACTTGAAGCTCAGTGATGGTCGGCTGCGTG	GTCATGGTCTTTGTAGTCCTCGAGTGCTGACTCAGTGACTTCAACAGGC
hOGT(K550R)	CTTGGTACCGAGCTCGGATCCGCCACCATGGCGTCTTCCGTGG	ACCATCACTGAGCCTCAAGTCTTTTGGATGTTCATATGGTGG
	AGGCTCAGTGATGGTCGGCTGCGTGTAGGATATGTGA	GTCATGGTCTTTGTAGTCCTCGAGTGCTGACTCAGTGACTTCAACAGGC
hOGT(K996R)	CTTGGTACCGAGCTCGGATCCGCCACCATGGCGTCTTCCGTGG	TCTTTGCTTCCAGACTCTGCCACGAACTTTCTTCAGGTATTCT
	GAGTCTGGAAGCAAAGAATATCTAGCCCTCTGTTCAACACCAAACA	GTCATGGTCTTTGTAGTCCTCGAGTGCTGACTCAGTGACTTCAACAGGC
hOGT(K999R)	CTTGGTACCGAGCTCGGATCCGCCACCATGGCGTCTTCCGTGG	ATATTCTTTGCCTCCAGACTTTGCCACGAACTTTCTTCAGGT
	TCTGGAGGCAAAGAATATCTAGCCCTCTGTTCAACACCAAACA	GTCATGGTCTTTGTAGTCCTCGAGTGCTGACTCAGTGACTTCAACAGGC
hCUL5	TATAGGATCCATGGCGACGTCTAAT	GCGCTCTAGATTATGCCATATATAT
hCUL5 ΔCH	TATAGGATCCATGGCGACGTCTAATCTG	ACCAACTTCTTTAAGCTTTGCTTCAATTATA
	GCGCAAGCTTAAAGAAGTTGGTCAATATG	GCGCTCTAGATTATGCCATATATAT
hCUL5 ΔNEDD	TATAGGATCCATGGCGACGTCTAATCTG	GCGCTCTAGATGTTTTTCAAAATTTCTA
hCUL5 ΔCH+ΔNEDD	TATAGGATCCATGGCGACGTCTAATCTG	GCGCTCTAGATTTAAGCTTTGCTTCAAT
hCUL5 ΔCR1-3	TATAGGATCCAAACTTGAATTACCTTTG	GCGCTCTAGATTATGCCATATATAT
Mouse Ogt shRNA	5’-GATCCGCATAATCCTGATAAGTTTGACTCGAGTCAAACTTATCAGGATTATGCTTTTTT-3’	

Supplementary Table 3: Primers Sequences.
