# Supplementary material for: Cullin5 drives experimental asthma exacerbations by modulating alveolar macrophage antiviral immunity
Source: Nat Commun. 2024 Jan 4;15:252. doi: 10.1038/s41467-023-44168-0 (PMC10766641; doi:10.1038/s41467-023-44168-0)
Supplement: Supplementary file 3 — Description of Additional Supplementary Files [file 41467_2023_44168_MOESM3_ESM.pdf]

Description of Additional Supplementary Files

Supplementary Data 1: Differentially expressed genes in the HDM+PR8 vs. HDM groups

Supplementary Data 2: The detail genes names of Fig. 1k

Supplementary Data 3: Differentially expressed genes in the LysMcre CUL5fl/fl HDM+PR8 vs. CUL5fl/fl HDM+PR8 groups

Supplementary Data 4: Differentially expressed genes in the HDM vs. PBS groups
