# Supplementary material for: Cullin5 drives experimental asthma exacerbations by modulating alveolar macrophage antiviral immunity
Source: Nat Commun. 2024 Jan 4;15:252. doi: 10.1038/s41467-023-44168-0 (PMC10766641; doi:10.1038/s41467-023-44168-0)
Supplement: Supplementary file 8 — Reporting Summary [file 41467_2023_44168_MOESM8_ESM.pdf]

nature portfolio

Corresponding author(s): Lei Sun and Feng Qian

Last updated by author(s): Nov 7, 2023

Reporting Summary

Nature Portfolio wishes to improve the reproducibility of the work that we publish. This form provides structure for consistency and transparency in reporting. For further information on Nature Portfolio policies, see our Editorial Policies and the Editorial Policy Checklist.

Statistics

For all statistical analyses, confirm that the following items are present in the figure legend, table legend, main text, or Methods section.

n/a	Confirmed	
☐	☒	The exact sample size (*n*) for each experimental group/condition, given as a discrete number and unit of measurement
☐	☒	A statement on whether measurements were taken from distinct samples or whether the same sample was measured repeatedly
☐	☒	The statistical test(s) used AND whether they are one- or two-sided<br>*Only common tests should be described solely by name; describe more complex techniques in the Methods section.*
☒	☐	A description of all covariates tested
☐	☒	A description of any assumptions or corrections, such as tests of normality and adjustment for multiple comparisons
☐	☒	A full description of the statistical parameters including central tendency (e.g. means) or other basic estimates (e.g. regression coefficient) AND variation (e.g. standard deviation) or associated estimates of uncertainty (e.g. confidence intervals)
☐	☒	For null hypothesis testing, the test statistic (e.g. *F*, *t*, *r*) with confidence intervals, effect sizes, degrees of freedom and *P* value noted<br>*Give P values as exact values whenever suitable.*
☒	☐	For Bayesian analysis, information on the choice of priors and Markov chain Monte Carlo settings
☒	☐	For hierarchical and complex designs, identification of the appropriate level for tests and full reporting of outcomes
☒	☐	Estimates of effect sizes (e.g. Cohen's *d*, Pearson's *r*), indicating how they were calculated

Our web collection on statistics for biologists contains articles on many of the points above.

Software and code

Policy information about availability of computer code

Data collection	ELISA data were collected on a Varioskan Flash multi-mode microplate reader. All the qPCR data for RNA levels were collected on an ABI Prism 7500 system. The microscopic pictures were acquired with Leica TCS SP UV confocal laser scanning microscope (Leica, Wetzlar, Germany), IX73 inverted fluorescence microscope (Olympus Corporation, Japan), and BX53 upright fluorescence microscope (Olympus Corporation, Japan). Advanced volcano plots and KEGG enrichment scatter plots were generated using the OmicStudio tools at https://www.omicstudio.cn/tool, and were analyzed with OmicStudioClassic (1.22.0), OmicStudioKits (2.12.0), and ggplot2 (3.3.3) using R version 4.1.3. Potential ubiquitination sites were predicted using BDM-PUB bioinformatics tool. AMs and viral infection-related genes were get from the GeneCards database. Western/Gel Plots were collected from multicolor fluorescence and chemiluminescence imaging (Bio-Rad, CFX Opus 96). Airway hyperresponsiveness (AHR) was tested using flexiVent FX equipped with module 2 (flexiVent FX, SCIREQ Scientific Respiratory Equipment Inc., Montréal, Canada). FACS Data were recorded using LSRFortessa (Becton, Dickinson and Company, USA). The gene sequences were collected from NCBI database.
Data analysis	GraphPad Prism 9.0. ImageJ 64. R software version 4.1.2. FLOWJO 10. OmicStudio tools at https://www.omicstudio.cn/tool.R version 4.1.3.

For manuscripts utilizing custom algorithms or software that are central to the research but not yet described in published literature, software must be made available to editors and reviewers. We strongly encourage code deposition in a community repository (e.g. GitHub). See the Nature Portfolio guidelines for submitting code & software for further information.

Data

Policy information about availability of data

All manuscripts must include a data availability statement. This statement should provide the following information, where applicable:

- Accession codes, unique identifiers, or web links for publicly available datasets
- A description of any restrictions on data availability
- For clinical datasets or third party data, please ensure that the statement adheres to our policy

The authors declare that the data supporting the findings of this study are available within the article. The RNA-seq raw data are deposited in GEO database under the number GSE246601 [https://www.ncbi.nlm.nih.gov/geo/query/acc.cgi?acc=GSE246601]. Other data in the figures are deposited in Figshare with DOI number 10.6084/m9.figshare.24531463. Source data are provided with this paper.

Research involving human participants, their data, or biological material

Policy information about studies with human participants or human data. See also policy information about sex, gender (identity/presentation), and sexual orientation and race, ethnicity and racism.

Reporting on sex and gender	*Use the terms sex (biological attribute) and gender (shaped by social and cultural circumstances) carefully in order to avoid confusing both terms. Indicate if findings apply to only one sex or gender; describe whether sex and gender were considered in study design; whether sex and/or gender was determined based on self-reporting or assigned and methods used. Provide in the source data disaggregated sex and gender data, where this information has been collected, and if consent has been obtained for sharing of individual-level data; provide overall numbers in this Reporting Summary. Please state if this information has not been collected. Report sex- and gender-based analyses where performed, justify reasons for lack of sex- and gender-based analysis.*
Reporting on race, ethnicity, or other socially relevant groupings	*Please specify the socially constructed or socially relevant categorization variable(s) used in your manuscript and explain why they were used. Please note that such variables should not be used as proxies for other socially constructed/relevant variables (for example, race or ethnicity should not be used as a proxy for socioeconomic status). Provide clear definitions of the relevant terms used, how they were provided (by the participants/respondents, the researchers, or third parties), and the method(s) used to classify people into the different categories (e.g. self-report, census or administrative data, social media data, etc.) Please provide details about how you controlled for confounding variables in your analyses.*
Population characteristics	*Describe the covariate-relevant population characteristics of the human research participants (e.g. age, genotypic information, past and current diagnosis and treatment categories). If you filled out the behavioural & social sciences study design questions and have nothing to add here, write "See above."*
Recruitment	*Describe how participants were recruited. Outline any potential self-selection bias or other biases that may be present and how these are likely to impact results.*
Ethics oversight	*Identify the organization(s) that approved the study protocol.*

Note that full information on the approval of the study protocol must also be provided in the manuscript.

Field-specific reporting

Please select the one below that is the best fit for your research. If you are not sure, read the appropriate sections before making your selection.

☒ Life sciences ☐ Behavioural & social sciences ☐ Ecological, evolutionary & environmental sciences

For a reference copy of the document with all sections, see nature.com/documents/nr-reporting-summary-flat.pdf

Life sciences study design

All studies must disclose on these points even when the disclosure is negative.

Sample size	Sample size were chosen based on our prior experience as for in vivo experiments (Reference 18). No sample-size calculations were performed for in vitro experiments. They were performed in triplicate as little variation was observed resulting in small standard deviation and high signal-to-noise ratio.
Data exclusions	No data exclusions.
Replication	All experiments were independently repeated three times with similar results.
Randomization	Unless when restricted by genotype of the animals, in vivo animal experiment group were randomly assigned to different treatment groups. When restricted by genotype of the animals, CUL5fl/fl mice and LysMcre CUL5fl/fl mice were assigned to different groups. For in vitro experiments, all samples were completely randomized to treatment groups.

Blinding	For in vivo experiments, the analysis were not blinded because the genotype of the mice were previously known. For other experiments in this paper, the data collection and analysis were blinded.

Behavioural & social sciences study design

All studies must disclose on these points even when the disclosure is negative.

Study description	*Briefly describe the study type including whether data are quantitative, qualitative, or mixed-methods (e.g. qualitative cross-sectional, quantitative experimental, mixed-methods case study).*
Research sample	*State the research sample (e.g. Harvard university undergraduates, villagers in rural India) and provide relevant demographic information (e.g. age, sex) and indicate whether the sample is representative. Provide a rationale for the study sample chosen. For studies involving existing datasets, please describe the dataset and source.*
Sampling strategy	*Describe the sampling procedure (e.g. random, snowball, stratified, convenience). Describe the statistical methods that were used to predetermine sample size OR if no sample-size calculation was performed, describe how sample sizes were chosen and provide a rationale for why these sample sizes are sufficient. For qualitative data, please indicate whether data saturation was considered, and what criteria were used to decide that no further sampling was needed.*
Data collection	*Provide details about the data collection procedure, including the instruments or devices used to record the data (e.g. pen and paper, computer, eye tracker, video or audio equipment) whether anyone was present besides the participant(s) and the researcher, and whether the researcher was blind to experimental condition and/or the study hypothesis during data collection.*
Timing	*Indicate the start and stop dates of data collection. If there is a gap between collection periods, state the dates for each sample cohort.*
Data exclusions	*If no data were excluded from the analyses, state so OR if data were excluded, provide the exact number of exclusions and the rationale behind them, indicating whether exclusion criteria were pre-established.*
Non-participation	*State how many participants dropped out/declined participation and the reason(s) given OR provide response rate OR state that no participants dropped out/declined participation.*
Randomization	*If participants were not allocated into experimental groups, state so OR describe how participants were allocated to groups, and if allocation was not random, describe how covariates were controlled.*

Ecological, evolutionary & environmental sciences study design

All studies must disclose on these points even when the disclosure is negative.

Study description	*Briefly describe the study. For quantitative data include treatment factors and interactions, design structure (e.g. factorial, nested, hierarchical), nature and number of experimental units and replicates.*
Research sample	*Describe the research sample (e.g. a group of tagged Passer domesticus, all Stenocereus thurberi within Organ Pipe Cactus National Monument), and provide a rationale for the sample choice. When relevant, describe the organism taxa, source, sex, age range and any manipulations. State what population the sample is meant to represent when applicable. For studies involving existing datasets, describe the data and its source.*
Sampling strategy	*Note the sampling procedure. Describe the statistical methods that were used to predetermine sample size OR if no sample-size calculation was performed, describe how sample sizes were chosen and provide a rationale for why these sample sizes are sufficient.*
Data collection	*Describe the data collection procedure, including who recorded the data and how.*
Timing and spatial scale	*Indicate the start and stop dates of data collection, noting the frequency and periodicity of sampling and providing a rationale for these choices. If there is a gap between collection periods, state the dates for each sample cohort. Specify the spatial scale from which the data are taken*
Data exclusions	*If no data were excluded from the analyses, state so OR if data were excluded, describe the exclusions and the rationale behind them, indicating whether exclusion criteria were pre-established.*
Reproducibility	*Describe the measures taken to verify the reproducibility of experimental findings. For each experiment, note whether any attempts to repeat the experiment failed OR state that all attempts to repeat the experiment were successful.*
Randomization	*Describe how samples/organisms/participants were allocated into groups. If allocation was not random, describe how covariates were controlled. If this is not relevant to your study, explain why.*
Blinding	*Describe the extent of blinding used during data acquisition and analysis. If blinding was not possible, describe why OR explain why blinding was not relevant to your study.*

Did the study involve field work? ☐ Yes ☐ No

Field work, collection and transport

Field conditions	*Describe the study conditions for field work, providing relevant parameters (e.g. temperature, rainfall).*
Location	*State the location of the sampling or experiment, providing relevant parameters (e.g. latitude and longitude, elevation, water depth).*
Access & import/export	*Describe the efforts you have made to access habitats and to collect and import/export your samples in a responsible manner and in compliance with local, national and international laws, noting any permits that were obtained (give the name of the issuing authority, the date of issue, and any identifying information).*
Disturbance	*Describe any disturbance caused by the study and how it was minimized.*

Reporting for specific materials, systems and methods

We require information from authors about some types of materials, experimental systems and methods used in many studies. Here, indicate whether each material, system or method listed is relevant to your study. If you are not sure if a list item applies to your research, read the appropriate section before selecting a response.

Materials & experimental systems

n/a	Involved in the study	
☐	☒	Antibodies
☐	☒	Eukaryotic cell lines
☒	☐	Palaeontology and archaeology
☐	☒	Animals and other organisms
☒	☐	Clinical data
☒	☐	Dual use research of concern
☒	☐	Plants

Methods

n/a	Involved in the study	
☒	☐	ChIP-seq
☐	☒	Flow cytometry
☒	☐	MRI-based neuroimaging

Antibodies

Antibodies used

RIG-I Ab (4200S), MAVS Ab (83000S), Phospho-TBK1/NAK Ab (Ser172, 5483S), TBK1/NAK Ab (38066S), Phospho-IRF-3 Ab (Ser396, 29047S), IRF-3 Ab (4302S), β-actin Ab (4970T), SOCS3 Ab (52113S), SOCS2 Ab (2779), SOCS1 Ab (3950T), Ubiquitin Ab (3936S), F4/80 Ab (71299S), HA-Tag Ab (3724S), Myc-Tag Ab (2272S), and O-GlcNAc transferase Ab (24083S). These antibodies were purchased from Cell Signaling Technology (Danvers, MA, USA). O-GlcNAc transferase Ab (sc-74546), O-GlcNac Ab (sc-59623), and CUL5 Ab (sc373822) were purchased from Santa Cruz Biotechnology (Dallas, TX, USA). These antibodies were diluted at 1:200 for WB and 1:50 for IP/IF. Goat anti-rabbit IgG HRP Ab (AB_2307291) and goat anti-mouse IgG HRP Ab (AB_10015289) were purchased from Jackson ImmunoResearch, Inc (West Grove, PA, USA). An anti-Flag mIgG1 antibody was purchased from Genomeditech (Shanghai) Co., Ltd (GM-30726AB). The reagents used in flow cytometry were as follows: APC anti-mouse CD11c Ab (clone N418,117310), PE anti-mouse CD170 Ab (clone S17007L, 155506), FITC anti-mouse Ly6G Ab (clone 1A8, 127606), APC anti-Ly6G Ab (clone 1A8, 127614), anti-mouse CD16/32 Ab clone S17011E, 156603) were purchased from BioLegend.

Validation

All the antibodies are commercially available and validated by manufacturers. The indicated species and application can be accessed on the manufacturers' website. These antibodies validation have been confirmed on mice according to the product introductions.
The specific products links were as following:
RIG-I Ab https://www.cellsignal.cn/products/primary-antibodies/rig-i-d33h10-rabbit-mab/4200
MAVS Ab https://www.cellsignal.cn/products/primary-antibodies/mavs-e8z7m-rabbit-mab/83000
Phospho-TBK1/NAK Ab,Ser172 https://www.cellsignal.cn/products/primary-antibodies/phospho-tbk1-nak-ser172-d52c2-xp-rabbit-mab/5483
TBK1/NAK Ab https://www.cellsignal.cn/products/primary-antibodies/tbk1-nak-e8i3g-rabbit-mab/38066
Phospho-IRF-3 Ab, Ser396 https://www.cellsignal.cn/products/primary-antibodies/phospho-irf-3-ser396-d6o1m-rabbit-mab/29047
IRF-3 Ab https://www.cellsignal.cn/products/primary-antibodies/irf-3-d83b9-rabbit-mab/4302
β-actin Ab https://www.cellsignal.cn/products/primary-antibodies/b-actin-13e5-rabbit-mab/4970
SOCS3 Ab https://www.cellsignal.cn/products/primary-antibodies/socs3-d6e1t-rabbit-mab/52113
SOCS2 Ab https://www.cellsignal.cn/products/primary-antibodies/socs2-antibody/2779
SOCS1 Ab https://www.cellsignal.cn/products/primary-antibodies/socs1-a156-antibody/3950
Ubiquitin Ab https://www.cellsignal.cn/products/primary-antibodies/ubiquitin-p4d1-mouse-mab/3936
F4/80 Ab https://www.cellsignal.cn/products/primary-antibodies/f4-80-bm8-1-rat-mab/71299
HA-Tag Ab https://www.cellsignal.cn/products/primary-antibodies/ha-tag-c29f4-rabbit-mab/3724
Myc-Tag Ab https://www.cellsignal.cn/products/primary-antibodies/myc-tag-antibody/2272

O-GlcNAc transferase Ab	https://www.cellsignal.cn/products/primary-antibodies/ogt-d1d8q-rabbit-mab/24083
O-GlcNAc transferase Ab	https://www.scbt.com/p/o-glcnac-transferase-antibody-f-12?requestFrom=search
O-GlcNac Ab	https://www.scbt.com/p/o-glcnac-antibody-ctd110-6?requestFrom=search
CUL5 Ab	https://www.scbt.com/p/cul-5-antibody-f-6?requestFrom=search
Goat anti-rabbit IgG HRP Ab	https://www.jacksonimmuno.com/catalog/products/111-035-144
goat anti-mouse IgG HRP Ab	https://www.jacksonimmuno.com/catalog/products/115-035-003
anti-Flag mIgG1 antibody	https://www.ddxcell.cn/pro_details.php?class_id=102101118101103&id=1033
APC anti-mouse CD11c Ab	https://www.biolegend.com/en-us/products/apc-anti-mouse-cd11c-antibody-1813
PE anti-mouse CD170 Ab	https://www.biolegend.com/en-us/products/pe-anti-mouse-cd170-siglec-f-antibody-16372
FITC anti-mouse Ly6G Ab	https://www.biolegend.com/en-us/products/fitc-anti-mouse-ly-6g-antibody-4775
APC anti-Ly6G Ab	https://www.biolegend.com/en-us/products/apc-anti-mouse-ly-6g-antibody-6115
anti-mouse CD16/32 Ab	https://www.biolegend.com/en-us/products/trustain-fcx-plus-anti-mouse-cd16-32-antibody-17085

Eukaryotic cell lines

Policy information about cell lines and Sex and Gender in Research

Cell line source(s)	Human cervical carcinoma HeLa cell line (CCL-2), The human leukemia monocytic cell THP-1 stable cell line (TIB-202), and human embryonic kidney cell line HEK293T (CRL-11268) cell line were purchased from ATCC (Rockville, USA)
Authentication	None of the cell lines were authenticated in these studies, but low passage number cell lines were utilized.
Mycoplasma contamination	All the cell lines are mycoplasma-free. They have been tested for mycoplasma contamination regularly.
Commonly misidentified lines (See ICLAC register)	No commonly misidentified lines were used.

Palaeontology and Archaeology

Specimen provenance	*Provide provenance information for specimens and describe permits that were obtained for the work (including the name of the issuing authority, the date of issue, and any identifying information). Permits should encompass collection and, where applicable, export.*
Specimen deposition	*Indicate where the specimens have been deposited to permit free access by other researchers.*
Dating methods	*If new dates are provided, describe how they were obtained (e.g. collection, storage, sample pretreatment and measurement), where they were obtained (i.e. lab name), the calibration program and the protocol for quality assurance OR state that no new dates are provided.*

☐ Tick this box to confirm that the raw and calibrated dates are available in the paper or in Supplementary Information.

Ethics oversight	*Identify the organization(s) that approved or provided guidance on the study protocol, OR state that no ethical approval or guidance was required and explain why not.*

Note that full information on the approval of the study protocol must also be provided in the manuscript.

Animals and other research organisms

Policy information about studies involving animals; ARRIVE guidelines recommended for reporting animal research, and Sex and Gender in Research

Laboratory animals	6-8 weeks C57BL/6 WT mice were obtained from the SLAC Laboratory Animal Corporation (Shanghai, China). Cul5fl/fl mice on C57BL/6 background were developed by Biocytogen Pharmaceuticals (Beijing, China) through conditionally containing a loxP sequence flanking the fourth and fifth exons of Cul5. Cul5fl/fl mice hybridized with LysMCre mice (Jackson Laboratory, Bar Harbor, ME, 004781) to obtain LysMCre Cul5fl/fl mice. All mice were housed (4–5 animals per cage) at 18–23°C with 40–60% humidity under a 12/12 h light/dark cycle with ad libitum access to food and water and keep in SPF environment. The housing, breeding, and experimental protocols complied with the National Institutes of Health Guide for the Care and Use of Laboratory Animals, and were approved by the Biological Research Ethics Committee of Shanghai Jiao Tong University (approval number A2018075).
Wild animals	No wild animals were used in the study.
Reporting on sex	Reporting on sex has not been collected. We used both male and female mice in this studies. We didn't notice any significant difference during experiment.
Field-collected samples	The study did not involved Field-collected samples.

Ethics oversight | All procedures involving mice were approved by the Institutional Animal Care and Use Committee of Shanghai Jiao Tong University (A2018075)

Note that full information on the approval of the study protocol must also be provided in the manuscript.

Clinical data

Policy information about clinical studies
All manuscripts should comply with the ICMJEguidelines for publication of clinical research and a completedCONSORT checklist must be included with all submissions.

Clinical trial registration | *Provide the trial registration number from ClinicalTrials.gov or an equivalent agency.*

Study protocol | *Note where the full trial protocol can be accessed OR if not available, explain why.*

Data collection | *Describe the settings and locales of data collection, noting the time periods of recruitment and data collection.*

Outcomes | *Describe how you pre-defined primary and secondary outcome measures and how you assessed these measures.*

Dual use research of concern

Policy information about dual use research of concern

Hazards

Could the accidental, deliberate or reckless misuse of agents or technologies generated in the work, or the application of information presented in the manuscript, pose a threat to:

No	Yes	
☐	☐	Public health
☐	☐	National security
☐	☐	Crops and/or livestock
☐	☐	Ecosystems
☐	☐	Any other significant area

Experiments of concern

Does the work involve any of these experiments of concern:

No	Yes	
☐	☐	Demonstrate how to render a vaccine ineffective
☐	☐	Confer resistance to therapeutically useful antibiotics or antiviral agents
☐	☐	Enhance the virulence of a pathogen or render a nonpathogen virulent
☐	☐	Increase transmissibility of a pathogen
☐	☐	Alter the host range of a pathogen
☐	☐	Enable evasion of diagnostic/detection modalities
☐	☐	Enable the weaponization of a biological agent or toxin
☐	☐	Any other potentially harmful combination of experiments and agents

Plants

Seed stocks | *Report on the source of all seed stocks or other plant material used. If applicable, state the seed stock centre and catalogue number. If plant specimens were collected from the field, describe the collection location, date and sampling procedures.*

Novel plant genotypes | *Describe the methods by which all novel plant genotypes were produced. This includes those generated by transgenic approaches, gene editing, chemical/radiation-based mutagenesis and hybridization. For transgenic lines, describe the transformation method, the number of independent lines analyzed and the generation upon which experiments were performed. For gene-edited lines, describe the editor used, the endogenous sequence targeted for editing, the targeting guide RNA sequence (if applicable) and how the editor was applied.*

Authentication | *Describe any authentication procedures for each seed stock used or novel genotype generated. Describe any experiments used to assess the effect of a mutation and, where applicable, how potential secondary effects (e.g. second site T-DNA insertions, mosiacism, off-target gene editing) were examined.*

ChIP-seq

Data deposition

☐ Confirm that both raw and final processed data have been deposited in a public database such as GEO.

☐ Confirm that you have deposited or provided access to graph files (e.g. BED files) for the called peaks.

Data access links<br>*May remain private before publication.*	*For "Initial submission" or "Revised version" documents, provide reviewer access links. For your "Final submission" document, provide a link to the deposited data.*
Files in database submission	*Provide a list of all files available in the database submission.*
Genome browser session<br>(e.g. UCSC)	*Provide a link to an anonymized genome browser session for "Initial submission" and "Revised version" documents only, to enable peer review. Write "no longer applicable" for "Final submission" documents.*

Methodology

Replicates	*Describe the experimental replicates, specifying number, type and replicate agreement.*
Sequencing depth	*Describe the sequencing depth for each experiment, providing the total number of reads, uniquely mapped reads, length of reads and whether they were paired- or single-end.*
Antibodies	*Describe the antibodies used for the ChIP-seq experiments; as applicable, provide supplier name, catalog number, clone name, and lot number.*
Peak calling parameters	*Specify the command line program and parameters used for read mapping and peak calling, including the ChIP, control and index files used.*
Data quality	*Describe the methods used to ensure data quality in full detail, including how many peaks are at FDR 5% and above 5-fold enrichment.*
Software	*Describe the software used to collect and analyze the ChIP-seq data. For custom code that has been deposited into a community repository, provide accession details.*

Flow Cytometry

Plots

Confirm that:

☒ The axis labels state the marker and fluorochrome used (e.g. CD4-FITC).

☒ The axis scales are clearly visible. Include numbers along axes only for bottom left plot of group (a 'group' is an analysis of identical markers).

☒ All plots are contour plots with outliers or pseudocolor plots.

☒ A numerical value for number of cells or percentage (with statistics) is provided.

Methodology

Sample preparation	For BALF collection, a tracheostomy was performed and a cannula was inserted into trachea. The left lung was lavaged three times with 1 mL cold PBS via tracheal catheter to obtain bronchoalveolar lavage (BAL) fluid. Counted the total number of cells in BALF. BALF was centrifuged at 4 °C at 600×g for 10 min. Isolated cells were first stained using the Zombie UVTM Fixable Viability Kit (67-68-5, BioLegend, San Diego, CA, USA) and Fc-blocking anti-mouse CD16/32 antibody (156603) to exclude dead cells and non-specific binding. Then, cells were stained with flow antibodies for 45 min at 4 °C℃.
Instrument	Data were recorded using flow cytometry (LSRFortessa, Becton, Dickinson and Company, USA)
Software	Data were analyzed using the FlowJo software (version 10)
Cell population abundance	All analysis have been performed on a population that included between 1000-20000 single cell in BALF.
Gating strategy	Collect all cells from FSC-A/SSC-A gate, then collect the single live cells, finally collect AMs, CD11c+Siglec-F+; neutrophils (Neu), Ly6G+; and eosinophils (Eos), CD11c−Siglec-F+.

☒ Tick this box to confirm that a figure exemplifying the gating strategy is provided in the Supplementary Information.

Magnetic resonance imaging

Experimental design

Design type	*Indicate task or resting state; event-related or block design.*
Design specifications	*Specify the number of blocks, trials or experimental units per session and/or subject, and specify the length of each trial or block (if trials are blocked) and interval between trials.*
Behavioral performance measures	*State number and/or type of variables recorded (e.g. correct button press, response time) and what statistics were used to establish that the subjects were performing the task as expected (e.g. mean, range, and/or standard deviation across subjects).*

Acquisition

Imaging type(s)	*Specify: functional, structural, diffusion, perfusion.*
Field strength	*Specify in Tesla*
Sequence & imaging parameters	*Specify the pulse sequence type (gradient echo, spin echo, etc.), imaging type (EPI, spiral, etc.), field of view, matrix size, slice thickness, orientation and TE/TR/flip angle.*
Area of acquisition	*State whether a whole brain scan was used OR define the area of acquisition, describing how the region was determined.*

Diffusion MRI ☐ Used ☐ Not used

Preprocessing

Preprocessing software	*Provide detail on software version and revision number and on specific parameters (model/functions, brain extraction, segmentation, smoothing kernel size, etc.).*
Normalization	*If data were normalized/standardized, describe the approach(es): specify linear or non-linear and define image types used for transformation OR indicate that data were not normalized and explain rationale for lack of normalization.*
Normalization template	*Describe the template used for normalization/transformation, specifying subject space or group standardized space (e.g. original Talairach, MNI305, ICBM152) OR indicate that the data were not normalized.*
Noise and artifact removal	*Describe your procedure(s) for artifact and structured noise removal, specifying motion parameters, tissue signals and physiological signals (heart rate, respiration).*
Volume censoring	*Define your software and/or method and criteria for volume censoring, and state the extent of such censoring.*

Statistical modeling & inference

Model type and settings	*Specify type (mass univariate, multivariate, RSA, predictive, etc.) and describe essential details of the model at the first and second levels (e.g. fixed, random or mixed effects; drift or auto-correlation).*
Effect(s) tested	*Define precise effect in terms of the task or stimulus conditions instead of psychological concepts and indicate whether ANOVA or factorial designs were used.*

Specify type of analysis: ☐ Whole brain ☐ ROI-based ☐ Both

Statistic type for inference<br>(See Eklund et al. 2016)	*Specify voxel-wise or cluster-wise and report all relevant parameters for cluster-wise methods.*
Correction	*Describe the type of correction and how it is obtained for multiple comparisons (e.g. FWE, FDR, permutation or Monte Carlo).*

Models & analysis

n/a	Involved in the study
☐	☐ Functional and/or effective connectivity
☐	☐ Graph analysis
☐	☐ Multivariate modeling or predictive analysis

Functional and/or effective connectivity	*Report the measures of dependence used and the model details (e.g. Pearson correlation, partial correlation, mutual information).*
Graph analysis	*Report the dependent variable and connectivity measure, specifying weighted graph or binarized graph, subject- or group-level, and the global and/or node summaries used (e.g. clustering coefficient, efficiency, etc.).*
Multivariate modeling and predictive analysis	*Specify independent variables, features extraction and dimension reduction, model, training and evaluation metrics.*
