# Supplementary material for: Cullin5 drives experimental asthma exacerbations by modulating alveolar macrophage antiviral immunity
Source: Nat Commun. 2024 Jan 4;15:252. doi: 10.1038/s41467-023-44168-0 (PMC10766641; doi:10.1038/s41467-023-44168-0)

Figure 6a

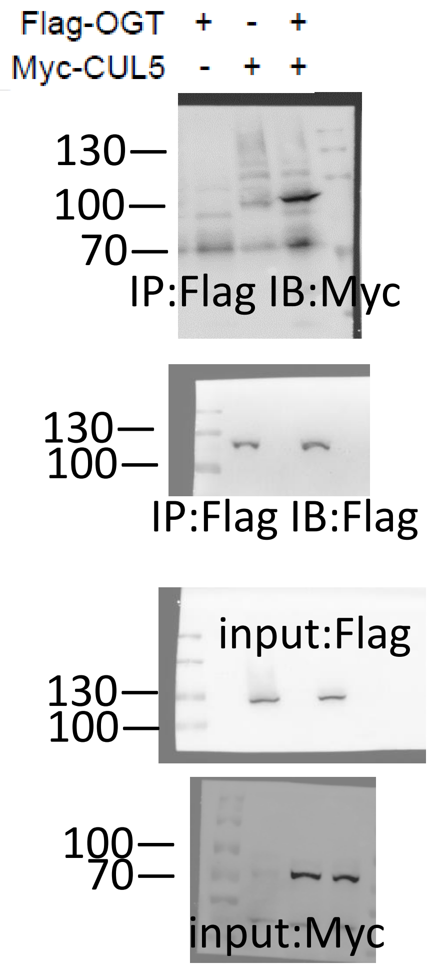

Figure 6 b

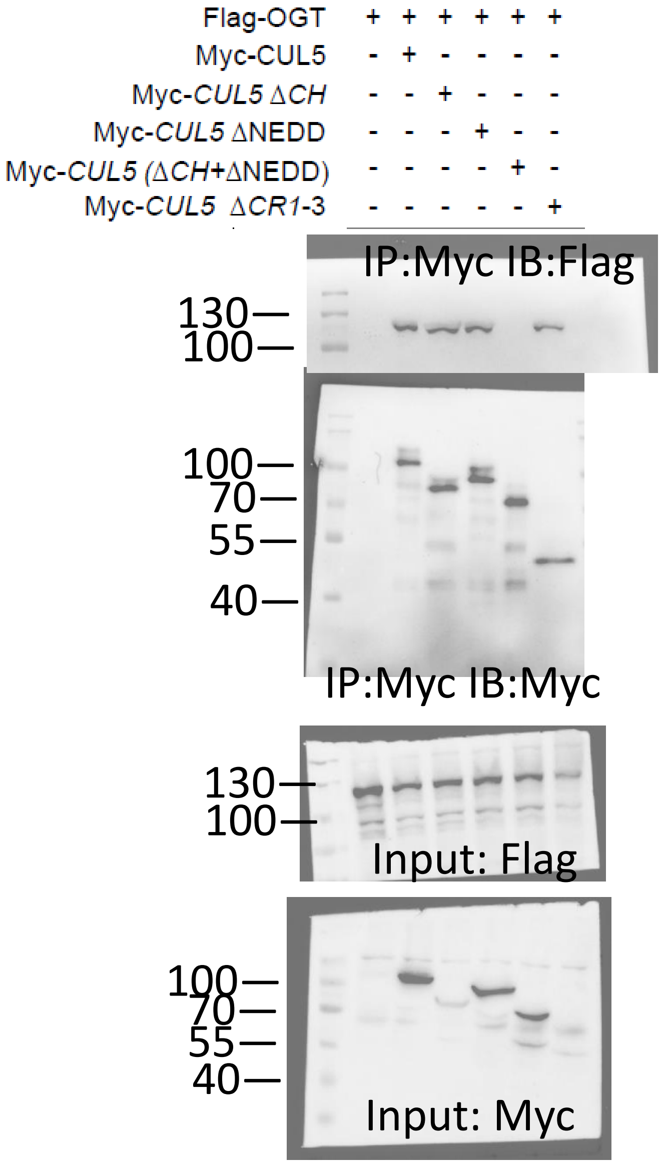

Figure 6 d

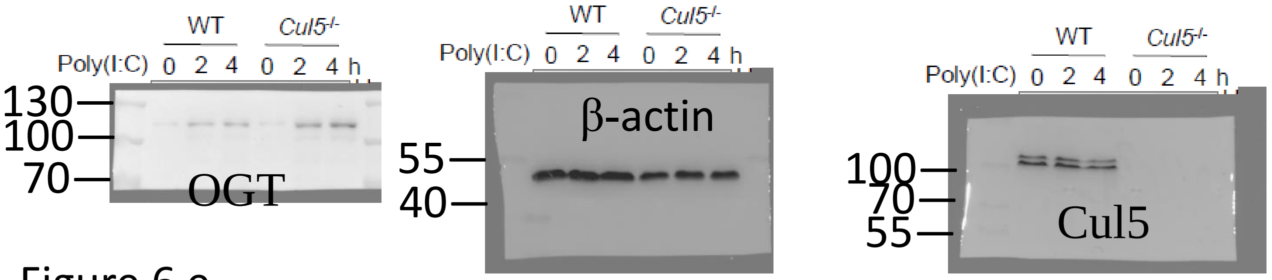

Figure 6 e

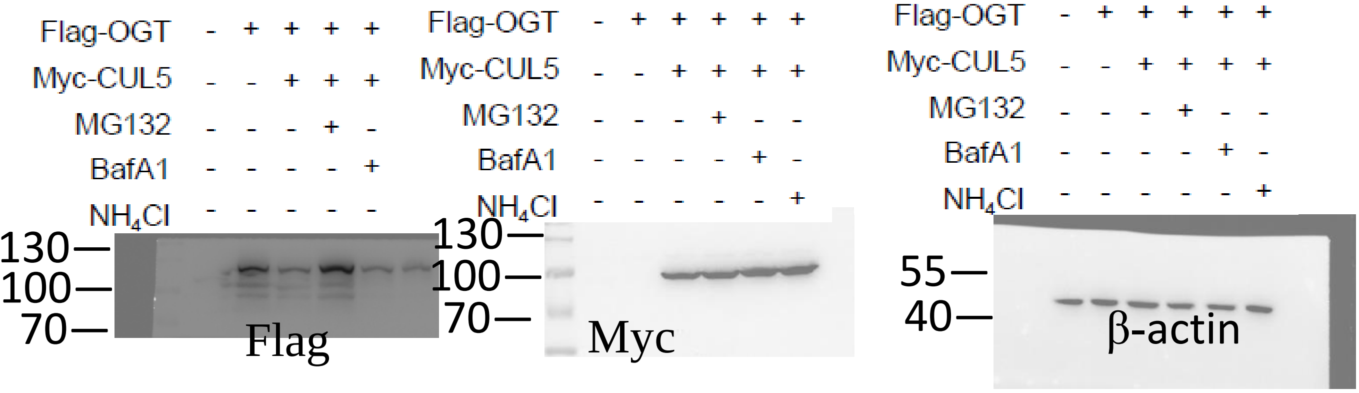

Figure 6f

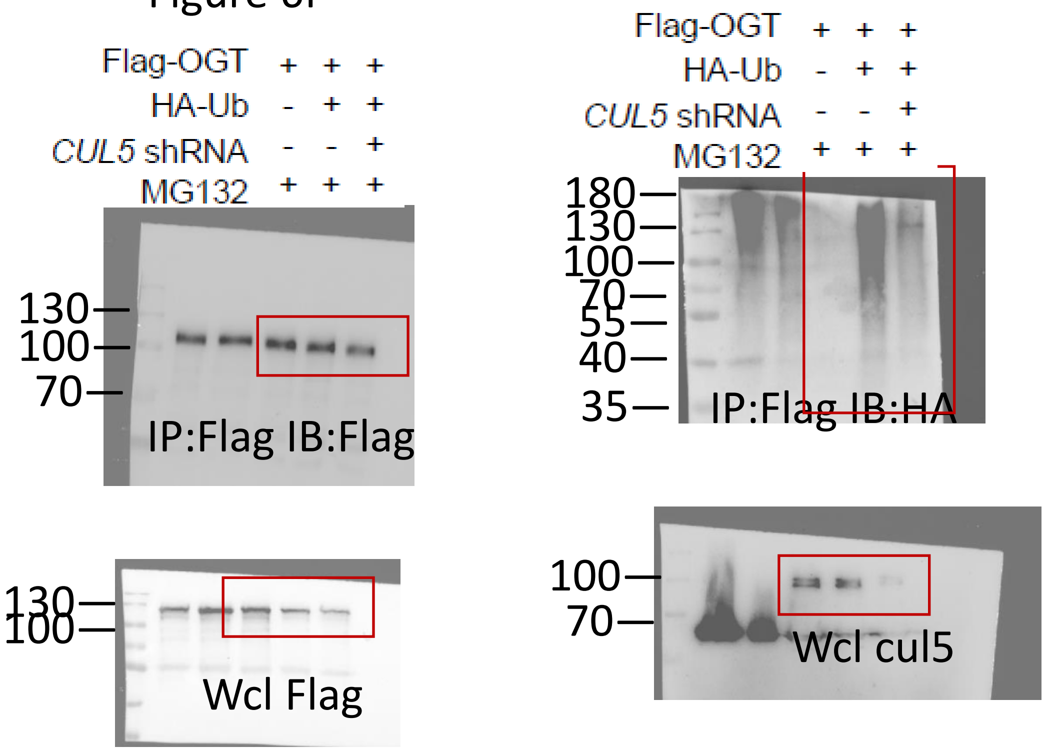

Figure 6g

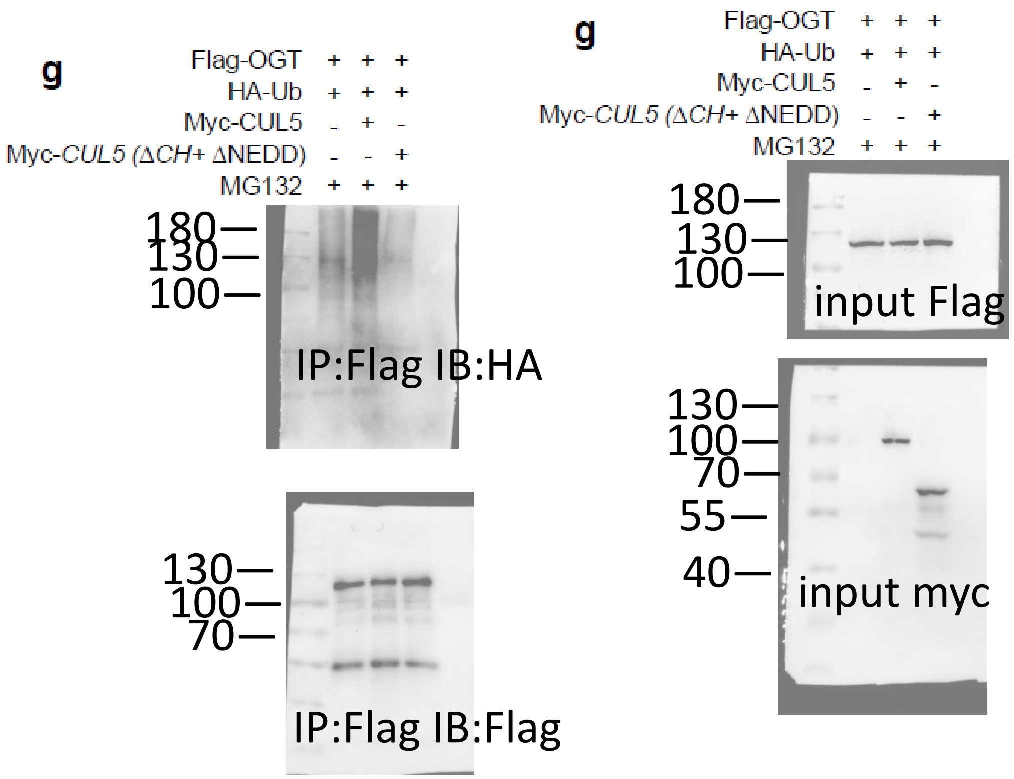

Figure 6h

Figure 6j

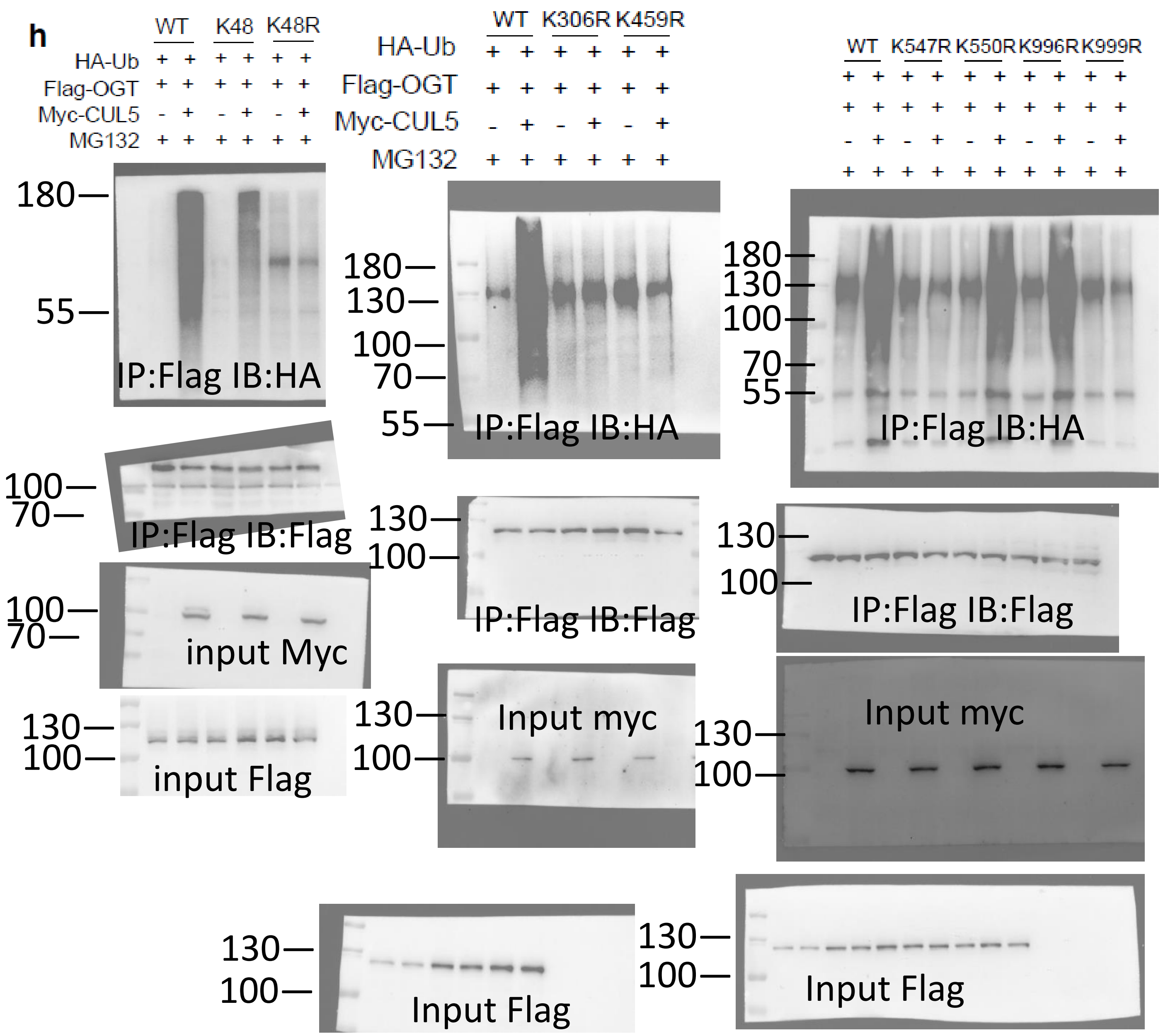

Figure 6l

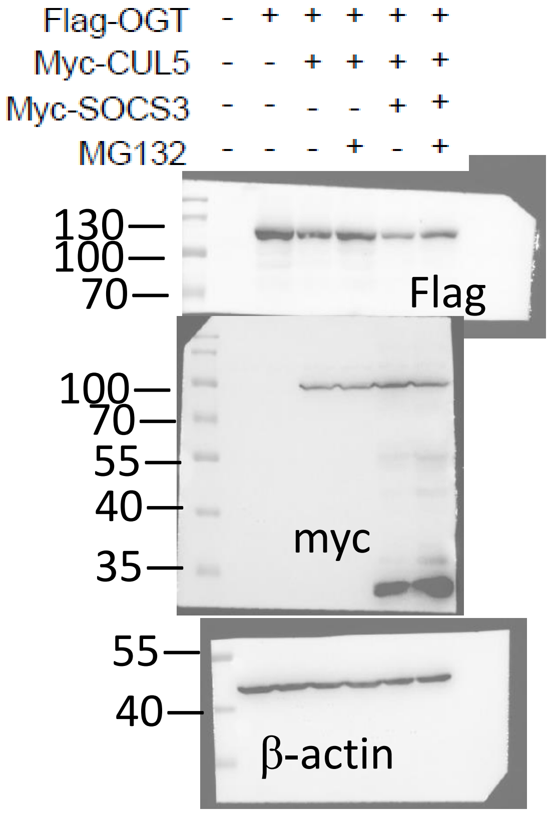

Figure 6m

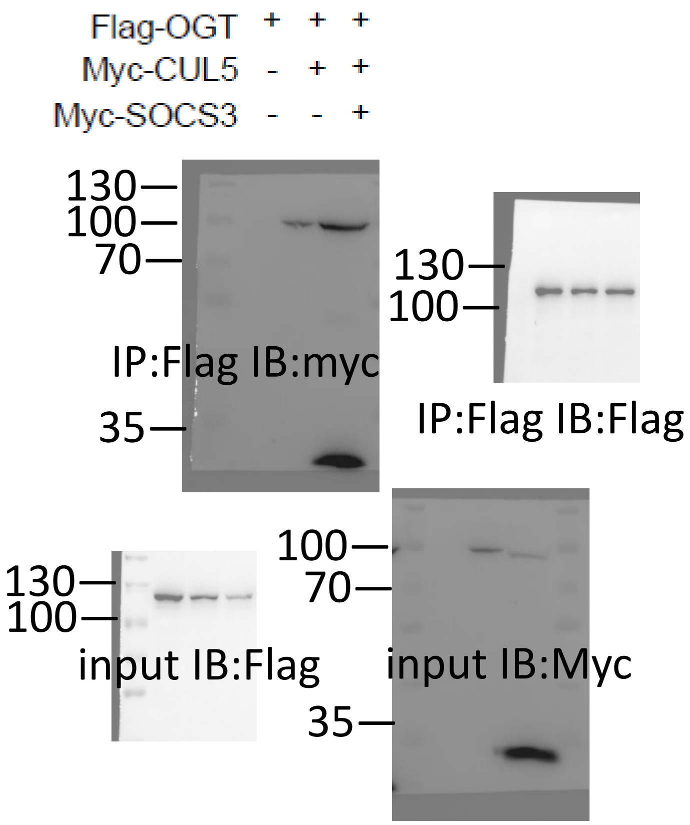

Figure 6n

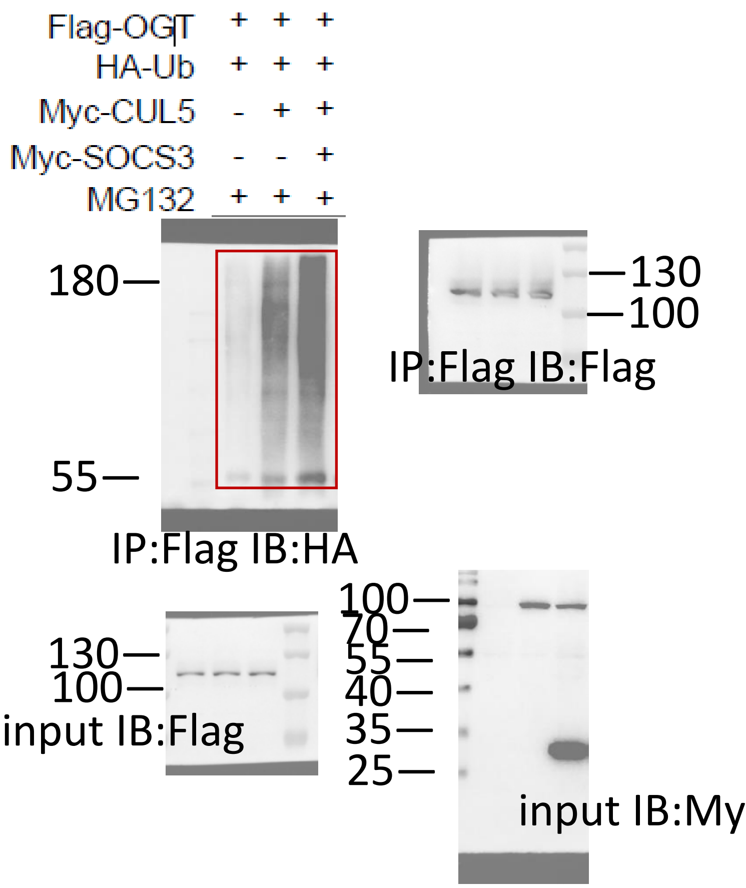

Supplement: Supplementary file 9 — Source Data [file 41467_2023_44168_MOESM9_ESM.zip › sun_sourcedata_Fig6.zip!/sun_sourcedata_Fig6.pdf]
