# Supplementary material for: Cullin5 drives experimental asthma exacerbations by modulating alveolar macrophage antiviral immunity
Source: Nat Commun. 2024 Jan 4;15:252. doi: 10.1038/s41467-023-44168-0 (PMC10766641; doi:10.1038/s41467-023-44168-0)
Supplement: Supplementary file 9 — Source Data [file 41467_2023_44168_MOESM9_ESM.zip › sun_sourcedata_Fig7.zip!/sun_sourcedata_Fig7.pdf]

Figure 7b

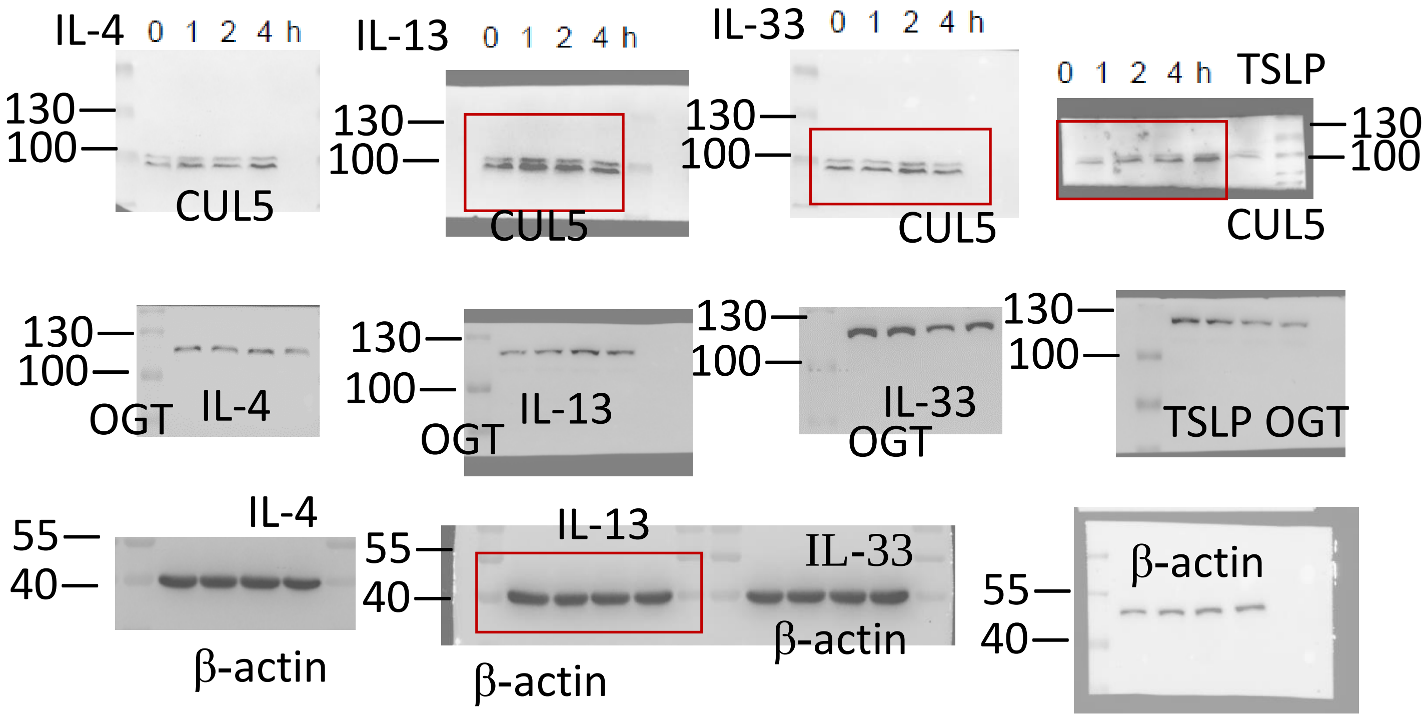

Figure 7c

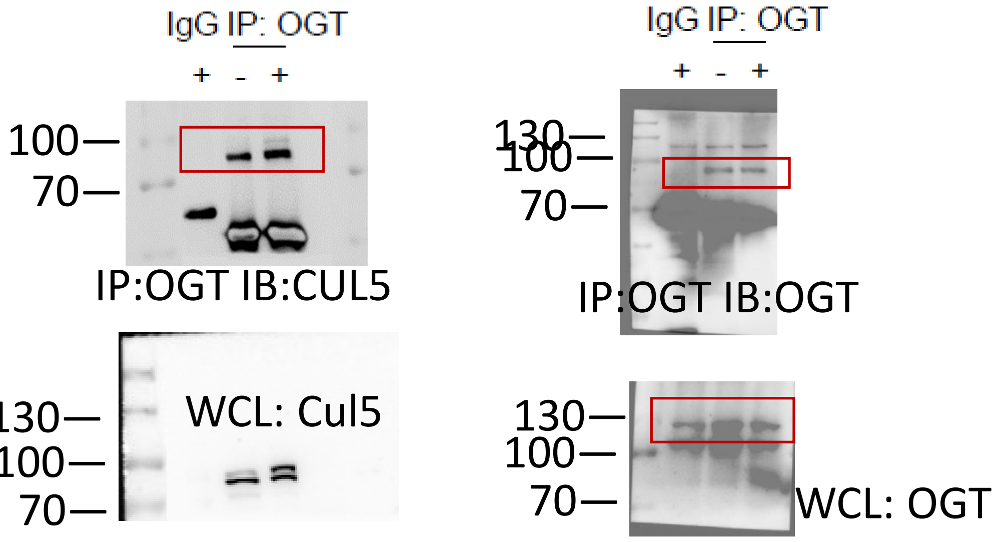

Figure 7g

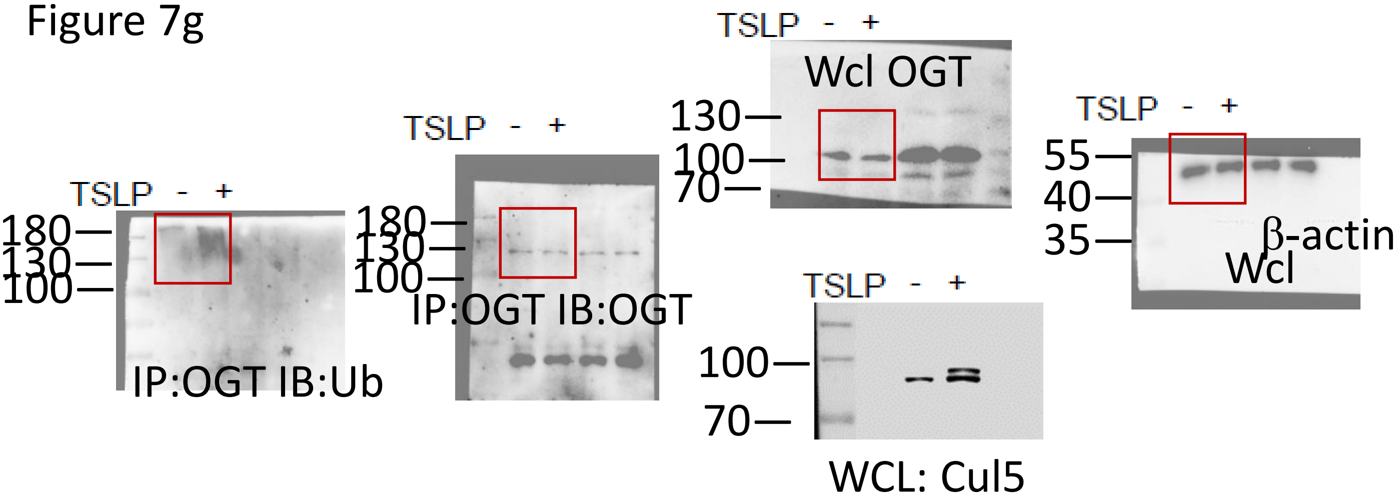

Figure 7h

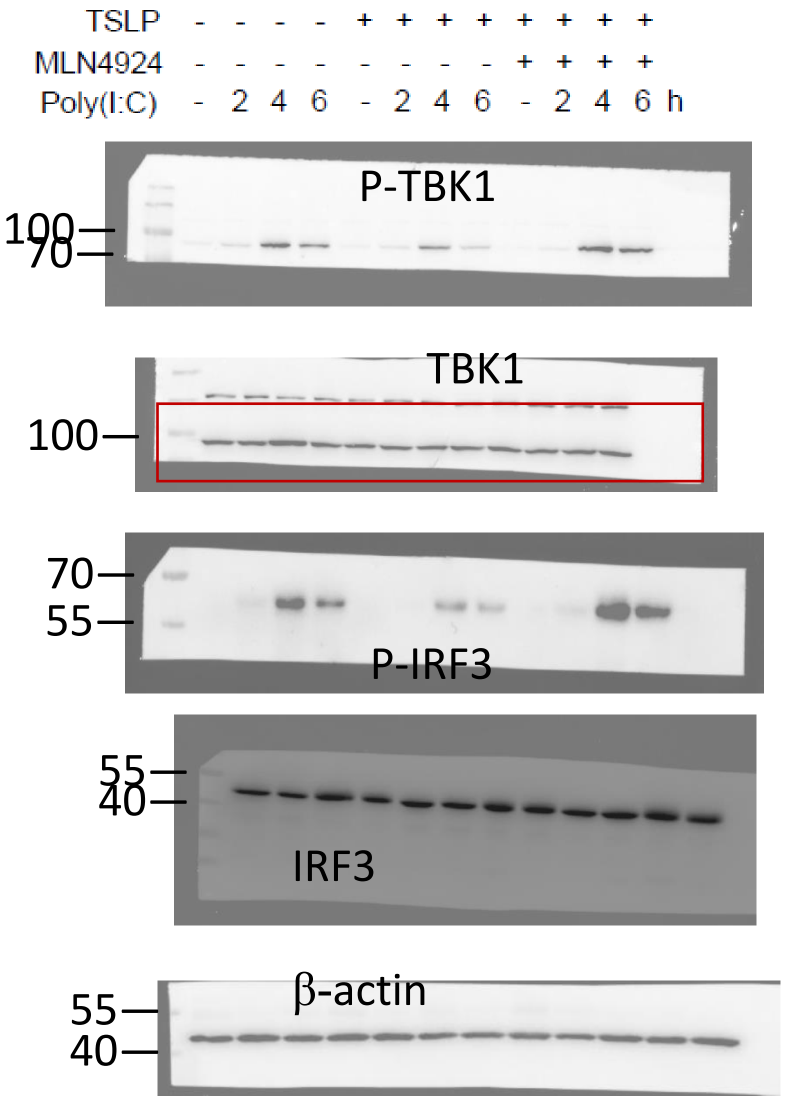

TSLP - - - - + + + + + + + +
MLN4924 - - - - - - - - + + + +
Poly(I:C) - 2 4 6 - 2 4 6 - 2 4 6 h
P-TBK1
100
70
TBK1
100
70
55
P-IRF3
55
40
IRF3
β-actin
55
40
