# Supplementary material for: Cullin5 drives experimental asthma exacerbations by modulating alveolar macrophage antiviral immunity
Source: Nat Commun. 2024 Jan 4;15:252. doi: 10.1038/s41467-023-44168-0 (PMC10766641; doi:10.1038/s41467-023-44168-0)

Supplementary Data Fig. 2b

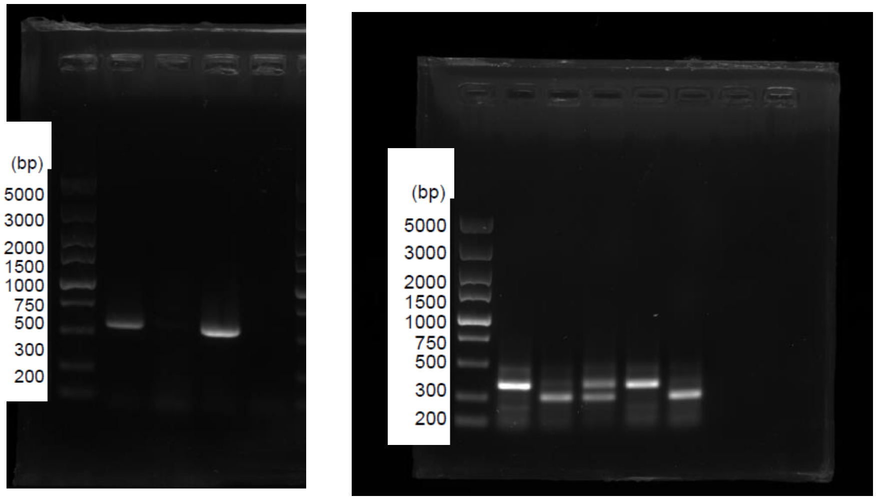

Supplementary Data Fig. 2c

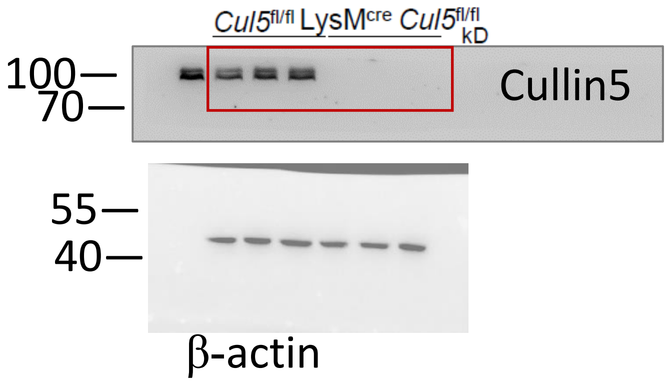

Supplement: Supplementary file 9 — Source Data [file 41467_2023_44168_MOESM9_ESM.zip › sun_sourcedata_supplemental_Fig2.zip!/sun_sourcedata_supplemental_Fig2.pdf]
